# Accumulating Progenitor Cells in the Luminal Epithelial Cell Layer Are Candidate Tumor Initiating Cells in a *Pten* Knockout Mouse Prostate Cancer Model

**DOI:** 10.1371/journal.pone.0005662

**Published:** 2009-05-22

**Authors:** Hanneke Korsten, Angelique Ziel-van der Made, Xiaoqian Ma, Theo van der Kwast, Jan Trapman

**Affiliations:** Department of Pathology, Josephine Nefkens Institute, Erasmus MC, Rotterdam, The Netherlands; Universität Heidelberg, Germany

## Abstract

The *PSA-Cre;Pten-loxP/loxP* mouse prostate cancer model displays clearly defined stages of hyperplasia and cancer. Here, the initial stages of hyperplasia development are studied. Immunohistochemical staining showed that accumulated pAkt^+^ hyperplastic cells overexpress luminal epithelial cell marker CK8, and progenitor cell markers CK19 and Sca-1, but not basal epithelial cell markers. By expression profiling we identified novel hyperplastic cell markers, including Tacstd2 and Clu. Further we showed that at young age prostates of targeted *Pten* knockout mice contained in the luminal epithelial cell layer single pAkt^+^ cells, which overexpressed CK8, Sca-1, Tacstd2 and Clu; basal epithelial cells were always pAkt^−^. Importantly, in the luminal epithelial cell layer of normal prostates we detected rare Clu^+^Tacstd2^+^Sca-1^+^ progenitor cells. These novel cells are candidate tumor initiating cells in *Pten* knockout mice. Remarkably, all luminal epithelial cells in the proximal region of normal prostates were Clu^+^Tacstd2^+^Sca-1^+^. However, in *PSA-Cre;Pten-loxP/loxP* mice, the proximal prostate does not contain hyperplastic foci. Small hyperplastic foci in prostates of *PSA-Cre;Pten-loxP/+* mice found at old age, showed complete *Pten* inactivation and a progenitor marker profile. Finally, we present a novel model of prostate development and renewal, including lineage-specific luminal epithelial progenitor cells. It is proposed that *Pten* deficiency induces a shift in the balance of differentiation to proliferation in these cells.

## Introduction

Prostate cancer is the most common tumor in men in countries with a western lifestyle, and a major cause of cancer-related mortality [1]. Complete *PTEN* inactivation is found in ∼20% of primary prostate tumors and in up to 60% of prostate cancer metastases [2]. Inactivation of one *PTEN* allele is even more common. High frequency of *PTEN* inactivation is found also in endometrium cancer and in glioblastoma [3]. Germ line mutations of *PTEN* are the cause of Cowden disease and Bannayan-Zonana syndrome, which are characterized by hamartomas and predisposition to breast and thyroid tumors [2], [3].

PTEN counteracts phosphoinositide-3-kinase (PI3K) signaling by balancing phosphatidylinositol (4,5)-phosphate (PIP2) and phosphatidylinositol (3,4,5)-phosphate (PIP3) levels in the cell [2]–[4]. PIP3 accumulation leads to phosphorylation of downstream targets, including AKT. As a consequence the activities of further downstream effectors are modulated and cell biological functions, including proliferation, apoptosis, cell size, polarity, metabolism, adhesion, migration and angiogenesis are changed [3]–[6]. Nuclear PTEN might play a role in maintaining genomic stability [7]. Moreover, it has recently been described that PTEN can control stem cell self-renewal [6], [8], [9].

Complete *Pten* inactivation in mice is embryonic lethal. *Pten+/−* mice are viable but develop several hyperplastic and dysplastic lesions in different organs [10], [11]. Conditional *Pten* knockout mouse models confirmed that *Pten* inactivation plays an important role in cancer development and tumor progression. Mice with prostate-specific *Pten* inactivation develop hyperplasia, mPIN and ultimately prostate cancer [12]–[15].

As first identified for hematopoietic cells, it is now generally accepted that all tissues contain rare tissue-specific stem cells that are capable of self-renewal and of differentiation through asymmetrical cell division [16], [17]. These cells are not only essential for organogenesis during development, but also for tissue renewal in the adult species. As clearly shown in different types of leukemia, tumors might develop by modification of hematopoietic stem cells or, alternatively, from multipotent or lineage-specific progenitor cells that have acquired stem cell-like characteristics [18]. Although less clear, the same mechanism has been proposed for the development of solid tumors, including prostate cancer. According to the stem cell model, each tumor contains a small number of cells with properties related to normal stem cells, that are essential for tumor maintenance [16], [19]–[21]. Complementary to the tumor stem cell theory, the clonal evolution model proposes that tumors can develop by expansion of dominant clones [22]–[24].

Study of tumor development in mouse prostate cancer models can be instrumental in understanding human prostate cancer. In the normal human and mouse prostate, stem cells and multipotent progenitor cells, or transit-amplifying cells, are proposed to be present in the basal epithelial cell layer [21], [25]–[27]. In addition, in mice, the proximal region of the prostate has been indicated as a putative stem/progenitor cell niche [28]–[30]. So far, our knowledge of initial steps in tumor development in mouse models of prostate cancer is limited. In the *Probasin(PB)-Cre* induced *Pten* knockout model, *Pten* inactivation in a p63^+^ stem/progenitor cell population in the basal epithelial cell layer has been postulated [31]. In a prostate-specific *Trp53/Rb* knockout model, luminal/neuroendocrine progenitor cells in the proximal prostate have been indicated as potential tumor initiating cells [32].

In the present study we investigated early steps in prostate tumor development in a different targeted *Pten* inactivation model, based on *PSA-Cre* expression. Previously, we described that in this model clearly defined stages of prostate hyperplasia and cancer can be discriminated [13]. Here we showed that hyperplastic cells in *Pten* knockout mice have a phenotype of luminal epithelial progenitor cells, including overexpression of CK8, CK19 and Sca-1. By expression profiling novel hyperplastic cell markers were identified. The first hyperplastic pAkt^+^ cells in prostates of young *Pten* knockout mice were found in the luminal epithelial cell layer. Importantly, we also identified at low frequency novel lineage-specific progenitor cells in the luminal epithelial cell layer of normal prostates. These cells might represent earlier postulated luminal intermediate/transit-amplifying cells [33], [34]. Our findings indicate that *Pten* inactivation in this mouse model leads to accumulation of the novel identified luminal epithelial progenitor cells by a drastic change of the differentiation/proliferation balance of these cells. Although all cells in the luminal epithelial cell layer in the proximal prostate showed expression of the novel lineage-specific markers, hyperplastic foci did not develop from this region of the prostate.

## Results

### Hyperplastic cells in prostates of PSA-Cre;Pten-loxP/loxP mice have a phenotype of luminal epithelial cells and express epithelial progenitor cell markers

Previously, we described prostate cancer development in *PSA-Cre;Pten-loxP/loxP* mice [13]. In prostates of 4–5 months (4–5m) old *PSA-Cre;Pten-loxP/loxP* mice hyperplastic epithelial cells overexpress Phospho-Akt (pAkt) and the luminal epithelial markers Cytokeratins 8/18 (CK8/18). Hyperplastic cells were negative for the basal epithelial markers Cytokeratins 5/14 (CK5/14). The androgen receptor (AR) was expressed at equal levels in normal and hyperplastic prostates [13].

In the present study we characterized by QPCR and immunohistochemistry hyperplastic prostate cells of *PSA-Cre;Pten-loxP/loxP* mice. First, the expression of additional progenitor, basal and luminal epithelial cell markers was analyzed at 2m and at 4–5m. At these ages prostates of *PSA-Cre;Pten-loxP/loxP* mice are for ∼70% and >90% hyperplastic, respectively (Figure S1A). Expression of *Probasin* and *Nkx3.1*, markers of differentiated luminal cells, was much lower in hyperplastic prostates, but *CK8* was higher expressed in these tissues. Low expression of the basal epithelial cell markers *CK5* and *p63* was detected. Interestingly, expression of the epithelial progenitor cell marker *CK19* [35], [36] was high in hyperplastic prostates. *Nkx3.1*, *CK8*, *CK19* and *p63* mRNA expression data were confirmed by immunohistochemistry on normal and hyperplastic prostates (4–5m) (Figure S1B–S1I). Nuclear Nkx3.1 staining was seen in luminal epithelial cells of normal prostates, but nuclei of hyperplastic cells were hardly positive (Figure S1B–S1C). A faint CK8 staining was observed at the apical side of luminal epithelial cells in normal prostates, whereas in hyperplastic prostates of *PSA-Cre;Pten-loxP/loxP* mice CK8 was overexpressed (Figure S1D–S1E). Importantly, CK19 was clearly higher expressed in hyperplastic cells than in normal prostate epithelial cells (Fig. S1F–S1G). Hyperplastic cells were negative for the basal epithelial cell marker p63, but an apparent normal p63^+^ basal epithelial cell layer was present below a multilayer of hyperplastic cells (Figure S1H–S1I). From these data we conclude that *Pten* inactivation in prostates of *PSA-Cre;Pten-loxP/loxP* mice results in the accumulation of hyperplastic cells with luminal progenitor cell characteristics (Nkx3.1^+/−^CK8^+^CK19^+^p63^−^).

### Expression profiling identifies novel genes with high expression in hyperplastic prostate epithelium

**Figure 1:**
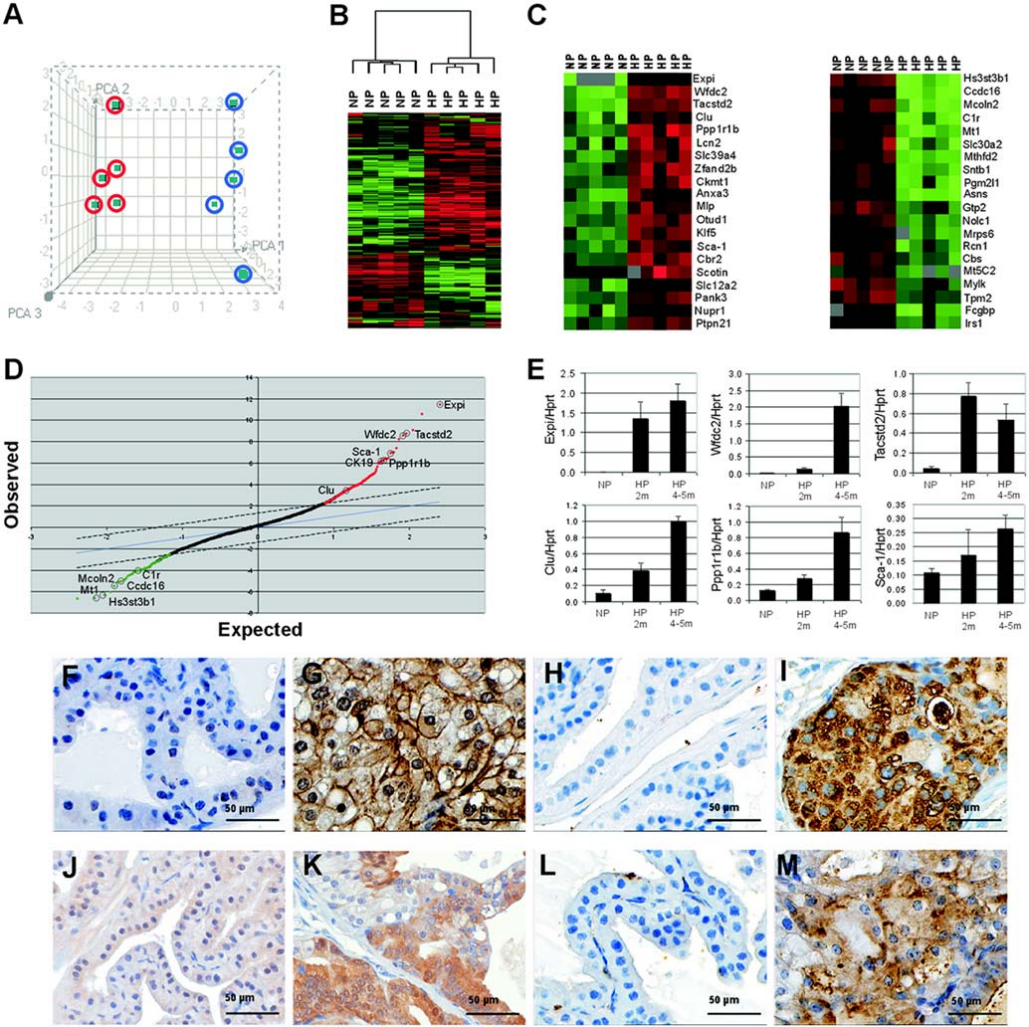
Identification of new hyperplastic cell markers in prostates of *PSA-Cre;Pten-loxP/loxP* mice. (A) Principal component analysis (PCA) of gene expression in normal prostates (blue circles) and hyperplastic prostates of *PSA-Cre;Pten-loxP/loxP* mice (red circles) at 4–5m. (B) Unsupervised hierarchical clustering of the gene expression profiles of five normal prostates (NP) and five hyperplastic prostates (HP). Green indicates lower gene expression and red indicates higher expression. (C) The twenty genes with the largest differential expression in HP as compared to NP as determined by calculation of the difference in mean expression level. (D) Significance Analysis of Microarrays (SAM) of the same samples as shown in C. Note that by SAM analysis essentially identical genes were identified as by calculation of the difference in mean expression level. (E) QPCR analysis of the five genes with the highest expression in HP as compared to NP and of *Sca-1*. Each subgroup was composed of five prostate samples. The expression levels in hyperplastic prostates of 2m and 4–5m old mice and in prostates of control littermates are shown as average expression level +/− SE relative to *Hprt* expression. (F–M) Immunohistochemical analysis of new hyperplastic cell markers in NP and HP of *PSA-Cre;Pten-loxP/loxP* mice (4–5m). (F) Tacstd2 NP, (G) Tacstd2 HP, (H) Clu NP, (I) Clu HP, (J) Ppp1r1b NP, (K) Ppp1r1b HP, (L) Sca-1 NP and (M) Sca-1 HP.

Expression profiling was performed to identify novel genes differentially expressed in hyperplastic prostates of *PSA-Cre;Pten-loxP/loxP* mice (4–5m). Principal Component Analysis (PCA) and unsupervised hierarchical clustering (Figure 1A and 1B) of the cDNA array data showed a clear differential gene expression profile between hyperplastic prostates and normal prostates.

To identify genes preferentially expressed in hyperplastic prostate cells, differences in mean expression levels were calculated. The mouse progenitor/stem cell marker *Sca-1* was one of the top twenty genes with the highest expression in hyperplastic prostates (Figure 1C); *CK19* was in the top fifty differentially expressed genes (data not shown). Genes that showed high expression in hyperplastic prostates by calculation of differences in mean expression level (Figure 1C), like *Expi, Wfdc2, Tacstd2 (Trop2), Clu, Ppp1r1b*, *Sca-1* and *CK19*, were also found overexpressed by Significance Analysis of Microarray (SAM) (Figure 1D). Full names of the top twenty overexpressed genes are listed in Table S1. A more extensive list of all significantly upregulated or downregulated genes identified by SAM analysis is provided in Table S2. Note that *CK19* is in the top twenty of upregulated genes.

The expression profiles of the five genes with the highest overexpression in hyperplastic prostates of targeted *Pten* knockout mice, *Expi, Wfdc2, Tacstd2, Clu* and *Ppp1r1b* (Figure 1C), and of *Sca-1*, were verified by QPCR in prostates of *Pten* knockout mice and in normal prostates at 2m and at 4–5m (Figure 1E). QPCR showed that the expression of *Expi* and *Tacstd2* in *PSA-Cre;Pten-loxP/loxP* mice was already high at 2m; *Clu* and *Sca-1* showed a gradually increasing expression level. However, for *Wfdc2* and *Ppp1r1b* the sharpest raise in expression was observed in completely hyperplastic prostates (4–5m).

Next, expression of markers for which appropriate antibodies were available, Tacstd2, Clu, Ppp1r1b and Sca-1, was studied by immunohistochemistry on normal and hyperplastic prostates (Figure 1F–1M). Tacstd2 immunohistochemistry showed membrane staining in hyperplastic tissues, in agreement with its known location as a transmembrane protein [37]. Clu was mainly present in the cytoplasm of hyperplastic cells. As predicted from the QPCR data, the expression pattern of Ppp1r1b in hyperplastic cells was more heterogeneous. In agreement with the CK19 staining (Figure S1), Sca-1 staining indicated that hyperplastic cells have a progenitor cell phenotype.

Genes with lower expression in hyperplastic prostates of *PSA-Cre;Pten-loxP/loxP* mice were also identified both by calculation of the difference in mean expression level and by SAM (Figure 1C and 1D). The expression profiles of genes with the lowest expression in hyperplastic prostates, *Hs3st3b1, Ccdc16, Mcoln2, C1r* and *Mt1* (Figure 1C), were confirmed by QPCR (Figure S2). Expression of all genes was already low in hyperplastic prostates at 2m in *PSA-Cre;Pten-loxP/loxP* mice.

### Initial pAkt^+^ hyperplastic cells in the luminal epithelial cell layer in prostates of PSA-Cre;Pten-loxP/loxP mice express Clu, Tacstd2 and Sca-1

**Figure 2:**
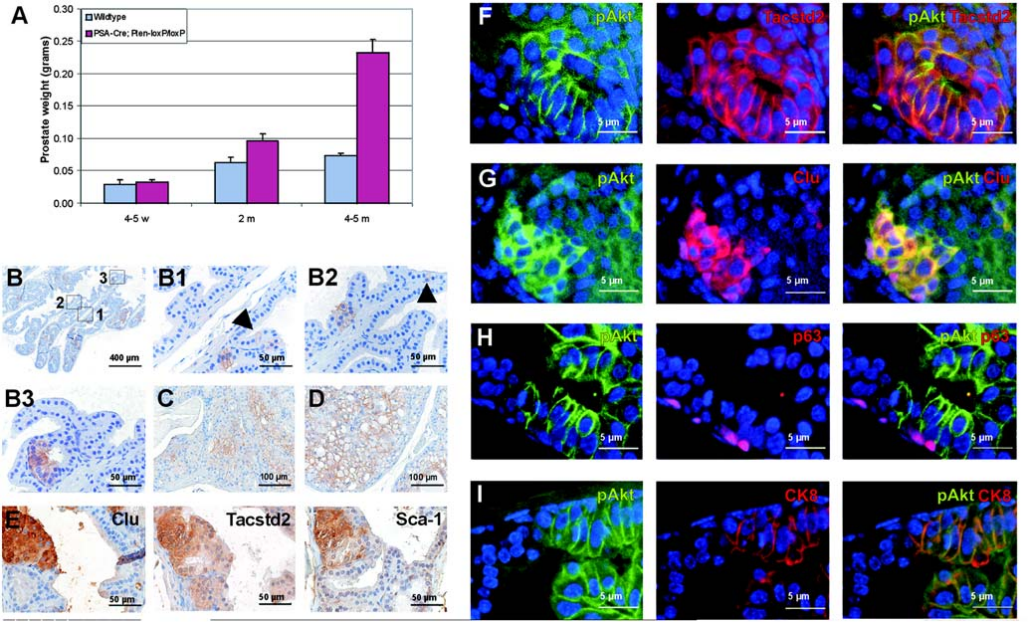
Single pAkt+ luminal epithelial cells in prostates of young *PSA-Cre;Pten-loxP/loxP* mice (4–5w) are Clu^+^Tacstd2^+^Sca-1^+^. (A) Prostate weights (average +/− SE) of wild type mice and *PSA-Cre;Pten-loxP/loxP* mice at 4–5w, 2m and 4–5m. (B) pAkt staining of hyperplastic foci/cells in the luminal epithelial cell layer of a prostate of a 4–5w old *PSA-Cre;Pten-loxP/loxP* mouse. Magnifications of three indicated regions are shown in B1, B2 and B3. Arrow heads in B1 and B2 indicate single pAkt^+^ cells. Phospho-Akt staining of prostates of 2m (C) and 4–5m (D) old *PSA-Cre;Pten-loxP/loxP* mice shows that respectively ∼70% and 100% of the luminal epithelial cells were pAkt^+^. (E) Consecutive slides of a prostate of a 4–5w old *PSA-Cre;Pten-loxP/loxP* mouse stained for Clu, Tacstd2 and Sca-1 shows coexpression of these markers in a small hyperplastic focus. Immunofluorescent double stainings confirmed co-localization of (F) Tacstd2 and pAkt, (G) Clu and pAkt and (I) CK8 and pAkt. (H) pAkt^+^ cells were not observed in the p63^+^ basal epithelial cell layer.

To collect information of candidate tumor initiating cells, we examined in detail the early development of hyperplasia in prostates of *PSA-Cre;Pten-loxP/loxP* mice, which starts at 4–5 weeks (4–5w). Prostates of wild type mice at 4–5w were half the size of those of adult mice (2m and 4–5m) (Figure 2A). In contrast to prostate weights of older mice (2m and 4–5m), prostate weights of young *PSA-Cre;Pten-loxP/loxP* mice (4–5w) were not different from control littermates (Figure 2A). At 4–5m the prostate weights of *PSA-Cre;Pten-loxP/loxP* mice were ∼3-fold higher than those of controls, caused by the increased number and size of hyperplastic cells.

Phospho-Akt expression was used as marker of *Pten* inactivation to visualize the first Pten negative cells. At 4–5w, scattered throughout all prostate lobes, single pAkt^+^ hyperplastic cells and small pAkt^+^ foci were detected (Figure 2B). Importantly, all pAkt^+^ cells were exclusively present in the luminal epithelial cell layer and not in the basal epithelial cell layer. At 2m and at 4–5m ∼70% and almost 100% of the luminal epithelial cells showed pAkt membrane staining, respectively (Figure 2C and 2D). Basal epithelial cells remained pAkt negative. Sca-1 and the new hyperplastic cell markers Clu and Tacstd2 were also expressed in the initial hyperplastic foci (Figure 2E), but Ppp1r1b expression could not yet be detected in hyperplastic cells at 4–5w (data not shown).

To allow accurate characterization of initial hyperplastic pAkt^+^ cells, immunofluorescent double staining was carried out (Figure 2F–2I). Importantly, all pAkt^+^ cells showed expression of Clu and Tacstd2. Moreover, all pAkt^+^ cells overexpressed CK8 and were negative for the basal epithelial cell marker p63. This finding strongly suggests that the first pAkt^+^ cells, which were exclusively observed in the luminal epithelial layer, are identical to the majority of accumulating hyperplastic cells with an epithelial progenitor cell phenotype at 4–5m.

### PSA-Cre;Pten-loxP/+ mice develop hyperplastic foci with the same marker profile as hyperplastic foci in prostates of PSA-Cre;Pten-loxP/loxP mice

Previously, we reported that heterozygous *PSA-Cre;Pten-loxP/+* mice do not develop prostate tumors, but that they can develop hyperplastic foci at older age [13]. The availability of novel hyperplastic cell markers allowed a more accurate study of hyperplasia development in these mice. Clu staining of prostates of *PSA-Cre;Pten-loxP/+* mice showed that already at 4–5m a few small hyperplastic foci could be detected (Figure S3). At 7–8m the number of hyperplastic foci was still very low, but a clear increase of hyperplastic foci was detected in older mice (>11m). Clu^+^ hyperplastic foci were not observed in prostates of control littermates (data not shown).

**Figure 3:**
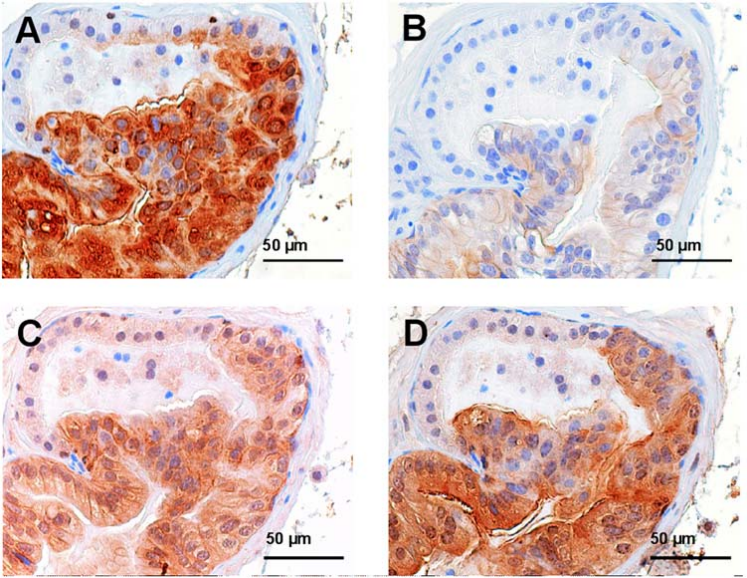
Hyperplastic cells in prostates of *PSA-Cre;Pten-loxP/+* mice and *PSA-Cre;Pten-loxP/loxP* mice express identical markers. Consecutive sections of a hyperplastic focus in the prostate of a *PSA-Cre;Pten-loxP/+* mouse were stained for (B) pAkt and the hyperplastic cell markers (A) Clu, (C) Tacstd2 and (D) Sca-1 by immunohistochemistry.

Interestingly, like in prostates of young *PSA-Cre;Pten-loxP/loxP* mice hyperplastic Clu^+^ foci of *PSA-Cre;Pten-loxP/+* mice (Figure 3A) showed pAkt overexpression (Figure 3B). In line with this observation Pten staining was negative in these foci (data not shown), indicating that the second *Pten* allele was inactivated in the pAkt^+^ hyperplastic cells. Hyperplastic cells in *PSA-Cre;Pten-loxP/+* mice were also Tacstd2 and Sca-1 positive (Figure 3C and 3D). So, hyperplastic cells in prostates of *PSA-Cre;Pten-loxP/+* mice had an identical expression profile as hyperplastic foci in young *PSA-Cre;Pten-loxP/loxP* mice.

### Single Clu^+^Tacstd2^+^Sca-1^+^ epithelial progenitor cells are present in the luminal epithelial cell layer of normal prostates

**Figure 4:**
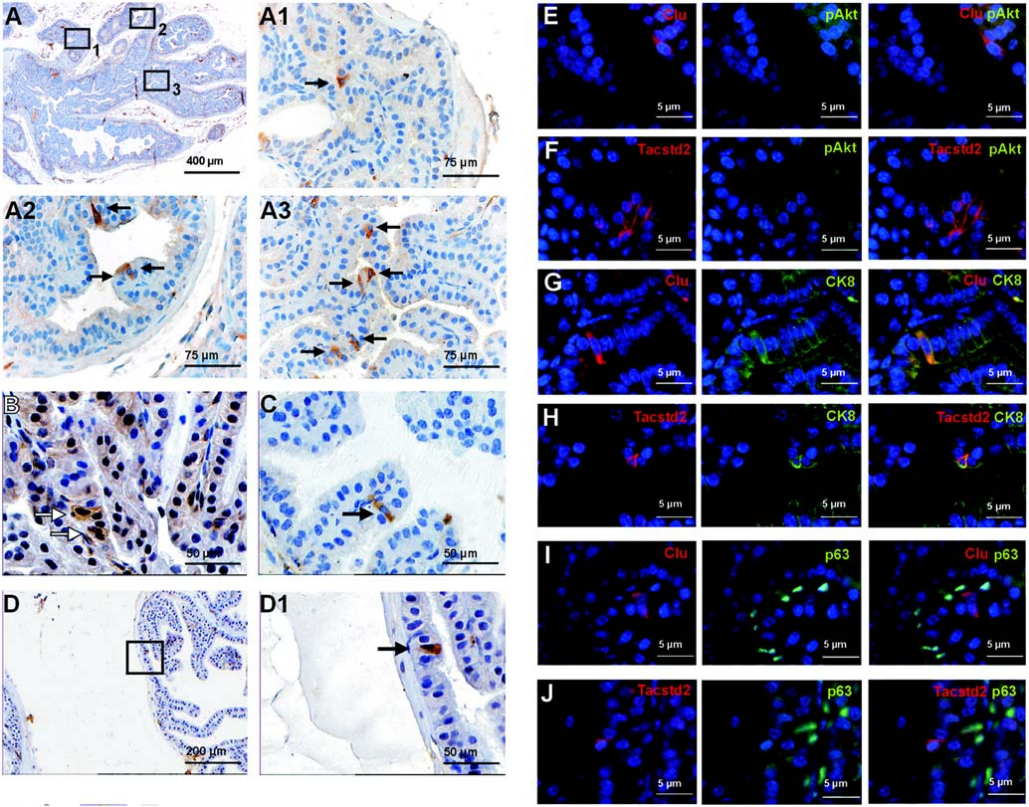
Single Clu^+^Tacstd2^+^Sca-1^+^ cells are present in the luminal epithelial cell layer of the normal prostate. (A) Clu staining of a normal prostate of a 4–5w old mouse. Scattered throughout the prostate lobe, single Clu^+^ cells in the luminal epithelial cell layer were observed. An overview of a whole prostate lobe and higher magnifications of three indicated regions are shown. Arrows indicate positive cells. (B) Tacstd2^+^ and (C) Sca-1^+^ cells in the luminal epithelial cell layer of the developing prostate (4–5w). (D) Clu staining of an adult normal prostate (4–5m) showed rare Clu^+^ cells in the luminal epithelial cell layer. (D1) Higher magnification of the indicated region in (D). (E–J) Immunofluorescent double staining of Clu^+^ and Tacstd2^+^ cells in the luminal epithelial cell layer of the normal prostate. In normal prostates Clu^+^ and Tacstd2^+^ cells were negative for pAkt (E,F), overexpressed CK8 (G,H) and did not express p63 (I,J).

Next, we investigated whether hyperplastic cell markers were expressed in normal prostates at different ages. Interestingly, at 4–5w single Clu^+^Tacstd2^+^Sca-1^+^ cells were detected in the luminal epithelial cell layer of normal prostates (Figure 4A–4C). Some basal epithelial cells stained positive for Tacstd2 and Sca-1, however, Clu^+^ cells were never observed in the basal epithelial layer. The presence of Clu^+^Tacstd2^+^Sca-1^+^ cells in the luminal epithelial cell layer of normal mouse prostates suggests that these cells are previously not yet identified lineage-specific progenitor cells of the luminal epithelial cells. It is tempting to speculate that in *Pten* knockout mice hyperplastic prostate cells with similar characteristics as these progenitor cells originate from these cells. This hypothesis is in line with the observation that pAkt^+^ hyperplastic cells were exclusively found in the luminal epithelial cell layer (Figure 2B, 2H, and 2I).

To estimate the frequency of Clu^+^Tacstd2^+^Sca-1^+^ epithelial progenitor cells at different ages, prostates from developing (4–5w) and from adult (4–5m) mice were stained for Clu expression. At 4–5w 1/250, and at 4–5m 1/25.000 luminal epithelial cells were Clu^+^, respectively (Figure 4A and 4D), indicating that in developing prostates the number of lineage-specific progenitor cells is much higher than in fully mature prostates. The presence of Clu^+^Tacstd2^+^Sca-1^+^ luminal progenitor cells in normal adult prostates makes these cells candidates from which hyperplastic foci develop in *PSA-Cre;Pten-loxP/+* mice, due to inactivation of the second *Pten* allele.

Clu^+^Tacstd2^+^ luminal epithelial progenitor cells in young normal prostates (4–5w) were further characterized by co-localization studies. pAkt overexpression in Clu^+^Tacstd2^+^ cells in the luminal epithelial cell layer of normal prostates was never observed (Figure 4E–4F). However, like hyperplastic cells in *PSA-Cre;Pten-loxP/loxP* mice, Clu^+^Tacstd2^+^ cells in the luminal epithelial cell layer of normal prostates overexpressed CK8 (Figure 4G–4H). Clu^+^ cells were exclusively found in the luminal epithelial cell layer, whereas Tacstd2^+^ cells were present in both the luminal and the basal epithelial cell layer (Figure 4G–4J, and Figure S4). Recently, high expression of Tacstd2 has been identified in basal epithelial cells of the proximal prostate, but not in basal epithelial cells in the more distal prostate [38].

### Hyperplastic foci do not develop from epithelial cells in the proximal prostate

**Figure 5:**
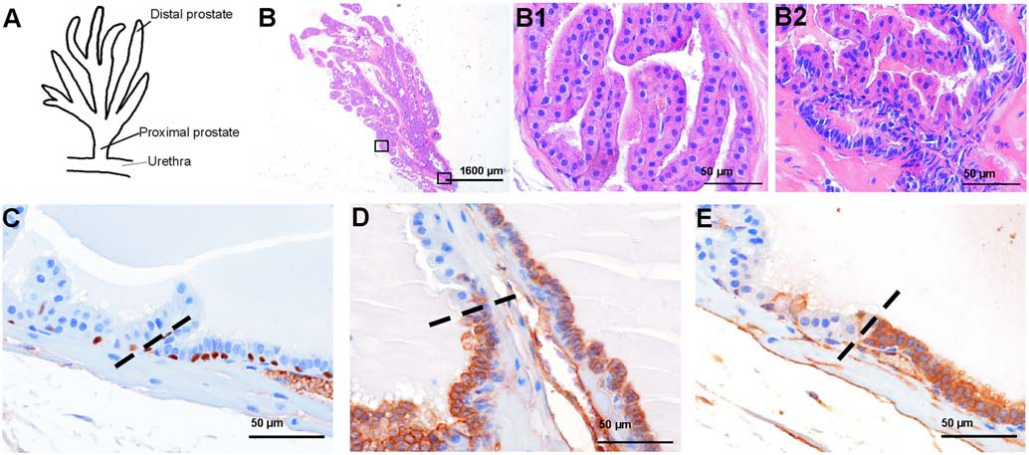
All luminal epithelial cells in the proximal prostate express luminal epithelial progenitor cell markers. (A) Schematic picture of a mouse prostate lobe indicating the urethra, the proximal and the distal prostate region. (B) Heamatoxylin eosin staining of a longitudinal positioned mouse prostate lobe. Magnifications of the distal (B1) and the proximal (B2) prostate, as indicated in B, showed a difference in morphology of the luminal epithelial cells in the proximal and the distal prostate. (C–E) Immunohistochemical analysis of luminal epithelial cells in the proximal region of a normal mouse prostate. (C) p63; (D) CK8 and (E) Sca-1 staining. Dashed lines indicate the abrupt transition of epithelium of the proximal to the distal prostate.

The proximal region of the mouse prostate has been proposed as a stem/progenitor cell niche [28]–[30]. A schematic view of the proximal and distal parts of a mouse prostate lobe is shown in Figure 5A. We studied the properties of the epithelial cells in the proximal prostate in more detail in *PSA-Cre;Pten-loxP/loxP* mice and in normal littermates.

As visualized in longitudinal sections of normal prostate lobes, luminal epithelial cells in the proximal prostate are more compact with less cytoplasm than luminal cells in distal parts of the prostate (Figure 5B). Like in the distal prostate, in the proximal prostate a p63^+^ basal epithelial cell layer was present below the luminal epithelial cells (Figure 5C). Interestingly, all luminal epithelial cells in the proximal prostate overexpressed CK8 (Figure 5D), like observed in rare lineage-specific luminal progenitor cells in the distal prostate and in hyperplastic cells in *PSA-Cre;Pten-loxP/loxP* mice. Sca-1 was also high expressed in proximal cells (Figure 5E), confirming that luminal epithelial cells in the proximal prostate have a luminal progenitor phenotype. As indicated by the interrupted line (Figure 5C–5E), there is an abrupt transition from epithelial cells with proximal characteristics to cells with properties of more distal luminal epithelial cells.

**Figure 6:**
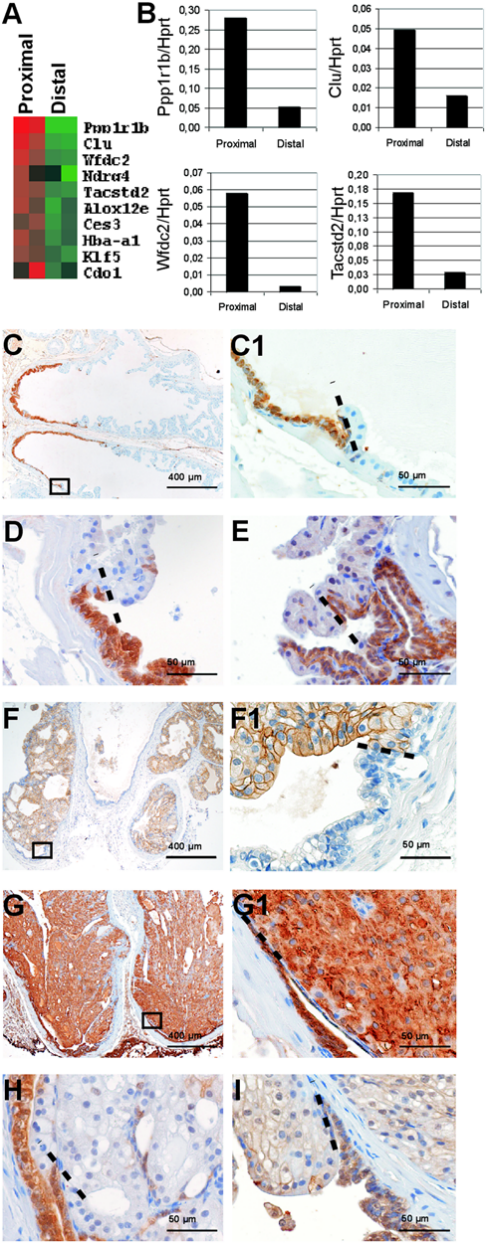
Hyperplastic prostates of *PSA-Cre;Pten-loxP/loxP* mice and luminal epithelial cells in the proximal prostate overexpress identical genes. (A) Expression profiling of the proximal and distal prostate region of a normal mouse prostate (4–5m) shows that genes high expressed in hyperplastic prostates of *PSA-Cre;Pten-loxP/loxP* mice are among the ten genes with the highest expression in two proximal prostates as compared to two distal prostates. (B) QPCR analysis of hyperplasia markers with high expression in the proximal prostate. (C–E) Immunohistochemical analysis confirms high expression of hyperplastic cell markers in the luminal epithelial cell layer of the proximal region of the normal prostate. (C) Clu staining of a normal prostate lobe, (C1) magnification of the transition of the proximal to the distal region as indicated in C. Staining for (D) Ppp1r1b and (E) Tacstd2. Hyperplasia development did not occur in the proximal prostate of *PSA-Cre;Pten-loxP/loxP* mice (4–5m). Proximal luminal epithelial cells of the hyperplastic prostates were negative for pAkt (F, magnification of transition proximal/distal prostate in F1), although these cells overexpressed (G, G1) Clu, (H) Ppp1r1b and (I) Tacstd2.

Next, expression profiling of proximal and distal regions of normal adult prostates was performed (Figure 6A). Strikingly, *Ppp1r1b*, *Clu*, *Wfdc2* and *Tacstd2* were among the genes with the highest expression in the proximal prostate. Expression array data were confirmed by QPCR (Figure 6B). As described above, these markers were also overexpressed in accumulated hyperplastic prostate cells of *PSA-Cre;Pten-loxP/loxP* mice (Figure 1) and in rare luminal progenitor cells in the distal prostate (Figure 4). Immunohistochemistry showed high Clu, Tacstd2 and Ppp1r1b expression in the luminal epithelial cells of the proximal prostate (Figure 6C–6E). The cDNA array data confirmed high expression of the luminal progenitor cell markers CK8 and Sca-1 in the proximal prostate (data not shown). In conclusion, a correlation was detected between the marker profile in hyperplastic prostates of *PSA-Cre;Pten-loxP/loxP* mice, rare lineage-specific progenitor cells in the distal prostate and the proximal luminal epithelial cells.

We investigated in *PSA-Cre;Pten-loxP/loxP* mice (4–5m) whether hyperplastic foci developed from luminal epithelial cells in the proximal prostate. Longitudinal sections of prostates showed that, although the distal prostate was completely hyperplastic, the epithelium in the proximal prostate was unaffected (Figure 6F–6I). Immunohistochemistry showed that, in contrast to the distal prostate, pAkt was not overexpressed in the proximal prostate (Figure 6F), indicating that *Pten* is not inactivated in this region of the prostate.

Further, we determined the marker profile of epithelial cells in urethral epithelium adjacent to the proximal prostate. In the superficial differentiated layer of the urothelium, the umbrella cells, high CK8 expression was observed. The highest expression of p63, Clu, Tacstd2, and Sca-1 was found in the intermediate/basal epithelial cell layers (Figure S5). The urethra data extend the prostate expression data indicating that Clu, Tacstd2 and Sca-1 show high expression in less differentiated epithelium.

## Discussion

In this study we defined the early stages of hyperplasia development in the *PSA-Cre;Pten-loxP/loxP* mouse prostate cancer model. Important aspects of early hyperplasia development and normal prostate development were sequentially addressed. We showed that: (i) Accumulating hyperplastic pAkt^+^ cells in prostates of *PSA-Cre;Pten-loxP/loxP* mice have a luminal epithelial cell phenotype with expression of known and novel identified markers of epithelial progenitor cells. (ii) The earliest single pAkt^+^ (Pten^−^) hyperplastic cells in the prostates of young targeted *Pten* knockout mice are exclusively present in the luminal epithelial cell layer. (iii) At low frequency, in the normal prostate, similar cells, but without pAkt overexpression, could be identified. (iv) Our data indicate further that *Pten* inactivation inhibits differentiation of luminal epithelial progenitor cells to mature cells. (v) We observed that the luminal epithelial cell layer of the proximal prostate is composed of cells that express the same markers as rare progenitor cells in more distal prostate regions. However, hyperplastic foci did not develop from the proximal prostate. (vi) Finally, we showed that at older age *PSA-Cre;Pten-loxP/+* mice developed in the prostate pAkt^+^ hyperplastic foci with an identical marker profile as in young *PSA-Cre;Pten-loxP/loxP* mice.

**Figure 7:**
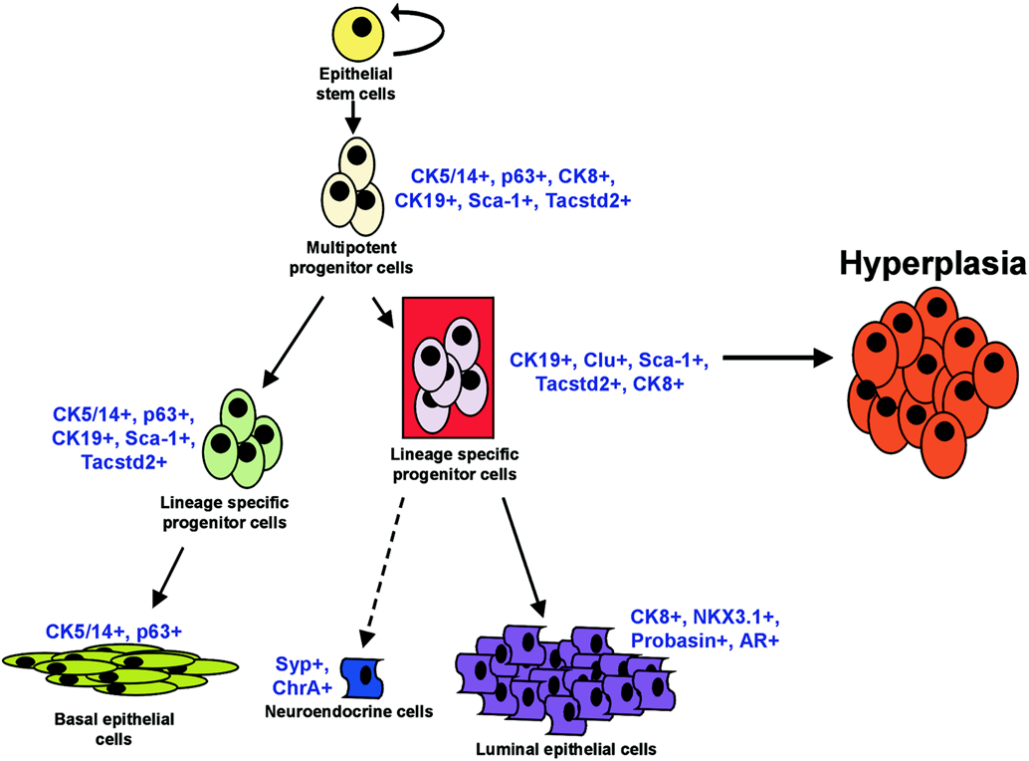
Model for hyperplasia development in *PSA-Cre;Pten-loxP/loxP* mice. The model shows novel identified lineage-specific luminal epithelial progenitor cells in the luminal epithelial cell layer as candidate tumor initiating cells in the prostate cancer mouse model, as indicated by a red background.

Our data accumulate into a hierarchical model of prostate renewal in which we define novel Clu^+^Tacstd2^+^Sca-1^+^ lineage-specific progenitor cells in the luminal epithelial layer of the normal prostate (Figure 7). We presume that, according to previous findings, prostate stem/multi-potent progenitor cells are situated in the basal epithelial cell layer [21], [25]–[27], [34], [39]. This cell layer might also contain lineage-specific progenitor cells of the basal epithelial cells. CK19 and Sca-1 positive cells are present both in the luminal and basal epithelial cell layer of the prostate [35], [36], but the expression of other epithelial cell markers differs in these cell populations. The CK19^+^CK8^+^Clu^+^Tacstd2^+^Sca1^+^ luminal epithelial progenitor cells as identified in this study are strong candidate tumor initiating cells in the *Pten* knockout prostate cancer model (Figure 7). This contrasts the opinion that prostate tumors should be derived from tissue stem cells or multipotent progenitor cells in the basal epithelial cell layer.

It is well-established that complete *Pten* inactivation in *Pten−/−* mice is embryonic lethal. These mice die at embryonic days 7–9, indicative for an important role of Pten in development. *Pten+/−* mice are viable, but develop during aging several tumor types, mostly T-cell lymphomas [10], [11]. During recent years, several mouse prostate cancer models based on *Pten* inactivation have been generated. In essentially all models, mice with prostate-specific *Pten* inactivation showed development of hyperplasia, mPIN lesions and ultimately prostate cancer [12]–[15]. Prostate hyperplasia in *Pten* knockout mice is characterized by cell enlargement, in line with the properties of Pten as regulator of cell size and protein synthesis [6], [40]. Accumulation of enlarged hyperplastic cells is unique for *Pten* inactivation in the prostate, and has not been seen in mouse prostate cancer models based on different genetic alterations [41]–[46].

Most studies in targeted *Pten* knockout prostate cancer models use mice with *Pten* inactivation by *PB-Cre* [14], [15], [31]. These mice develop hyperplasia early during prostate development, rapidly progressing to invasive tumors, accompanied by metastases [15], [31]. The slow model studied here, using Cre driven by the PSA promoter, induces clearly separate stages of hyperplasia and cancer progression [13]. Although the PSA promoter used is of human origin, it drives very specific transgene expression in the luminal epithelial cell layer of the mouse prostate [47].

Although hyperplastic Pten negative cells express AR [13], they are blocked in differentiation to mature cells, as deduced from the low expression of *Nkx3.1* and *Probasin* (Figure S1). So far, a role of Pten in differentiation of prostate luminal epithelial cells was unknown. Available data on the effect of *Pten* on differentiation in other systems is incomplete and conflicting. *Pten* deficiency was shown to stimulate neural stem cell proliferation without affecting differentiation [48], [49]. Mice with *Pten* inactivation in osteoblasts showed enhanced differentiation [50]. Based on the stimulation of cell proliferation by *PTEN* deficiency in many systems, it might be assumed that *Pten* inactivation stimulates proliferation of the accumulating progenitor cells, however, a high proliferation rate might also be an intrinsic property of these cells [13].

None of the genes with clear differential expression in hyperplastic prostates compared to normal prostates (Figure 1 and Figure S1) could be directly correlated to Pten signaling. Expression of known FOXO targets, one of the best studied downstream effectors of pAkt, was not clearly detected amongst the differentially expressed genes in hyperplastic prostates [51]. So, the differentially expressed genes are markers of the cell population enriched in hyperplastic prostates, indirectly induced by *Pten* inactivation.

The gene expression profile of the hyperplastic prostate presented in this study showed overlap with the gene expression profiles of prostate tumors of *PB-Cre;Pten-loxP/loxP* mice [15] and tumors from *PSA-Cre;Pten-loxP/loxP* mice (H. Korsten, unpublished). Out of the 50 genes with the highest expression in prostate tumors of *PB-Cre;Pten* knockout mice, 22 genes were present on our expression arrays. Almost all these genes showed higher expression in hyperplastic prostates of *PSA-Cre;Pten-loxP/loxP* mice. Five genes, *Expi, Clu, Anxa3, Tacstd2* and *Cbr2*, were among the top 20 genes with the highest expression in hyperplastic prostates (Figure 1C). Overexpression of genes in both hyperplasia and tumor indicates that altered expression of these specific genes is not sufficient for development of invasive tumors. A limited number of genes, including Col3a1, seems preferentially overexpressed in tumors [15]. These genes are candidate prostate tumor markers.

An important issue addressed in this study concerns the properties of the first altered cells. We scanned thoroughly prostates at 4–5w for cells overexpressing pAkt, as very sensitive marker for *Pten* inactivation. We never observed pAkt^+^ cells in the basal epithelial cell layer, although in the luminal epithelial cell layer single pAkt^+^ cells and small foci of pAkt^+^ cells were easily detected. High expression of hyperplasia markers Clu, Tacstd2 and Sca-1 was also found in the first hyperplastic cells in the luminal epithelial cell layer at 4–5w. Importantly, the Clu^+^Tacstd2^+^Sca-1^+^ cells, which also overexpress CK8, are at low frequency detectable in the luminal epithelial cell layer of normal prostates. Hence, we propose that these lineage-specific progenitor cells, as first identified in this study, are the target cells for *Pten* inactivation in the prostate cancer model (Figure 7). This would be in line with the properties of the PSA promoter/enhancer used to drive *Cre* expression, which is active in luminal epithelial cells of the mouse prostate [47] and in the more differentiated luminal epithelial cells in the human prostate. Our data indicate expansion of a rather homogeneous hyperplastic progenitor cell population induced by *Pten* inactivation as the first step in tumorigenesis. Follow-up studies, including orthotopic transplantation of hyperplastic cells in syngenic mice with a homogeneous genetic background should reveal further information on the mechanism of tumor development.

The newly identified lineage-specific Clu^+^Tacstd2^+^Sca-1^+^ progenitor cells in the luminal epithelial cell layer share characteristics with previously postulated luminal intermediate/transit-amplifying cells [33]–[36]. Multipotent transit-amplifying cells co-express basal epithelial cell and luminal epithelial cell markers, whereas the more differentiated intermediate/transit-amplifying cells in the luminal epithelial cell layer are negative for basal epithelial cell markers, but express CK8 and CK19 [35], [36]. It was proposed that the cell of origin for prostate cancer is a stem cell or transit-amplifying cell in the basal epithelial cell layer [33], [34]. Here, we show that progenitor cells with characteristics of intermediate/transient-amplifying cells in the luminal epithelial cell layer can function as tumor initiating cells in prostate cancer (Figure 7). Obviously, this does not exclude the possibility that stem cells and multipotent progenitor cells can have similar properties. This might depend on the properties of the gene that is activated or inactivated, and on the (promoter) system used to accomplish targeted gene activation or inactivation.

Our findings are substantially different from the *PB-Cre;Pten* knockout mouse model [31]. In these mice it was proposed that *Pten* inactivation in stem/multipotent progenitor cells in the basal epithelial cell layer is the initial event in prostate tumor development. In contrast to the *PSA-Cre;Pten* knockout mouse model, in this model proliferation of p63^+^ basal epithelial cells was observed. Hence, the modified *PB* promoter seems active in multipotent progenitor cells, whereas the *PSA* promoter/enhancer is active in lineage-specific luminal progenitor cells. Although comparison with human prostate cancer has its limitations, it should be noted that human prostate tumors do not show an increase in p63^+^ cells. In fact, negative staining for p63 is considered as indication of prostate cancer [52].

Although preliminary data are available [30], [53]–[55], further identification and characterization of candidate prostate stem cells and different types of progenitor cells is essential for unraveling prostate development and tumor initiation in mouse models. In normal mouse prostates a Sca-1^+^Integrinα6^+^ (CD49f) enriched cell population in the basal epithelial cell layer was reported to possess stem cell characteristics [38], [55]. Preliminary QPCR analysis of hyperplastic prostates of *PSA-Cre;Pten-loxP/loxP* mice indicated that integrinα6 (*CD49f*) mRNA expression was not substantially altered (H. Korsten, unpublished). However, we did find higher expression in hyperplastic prostates of *integrinα2 (CD49b)* (H. Korsten, unpublished), also observed to be overexpressed in a cell population enriched for stem/progenitor cells in the human prostate [56], [57]. Recently, another mouse prostate stem cell population was defined by a Sca-1^+^CD133^+^CD44^+^CD117^+^ phenotype [30]. This cell population showed low CK8 and higher p63 and CK14 expression. QPCR analysis showed a lower CD133 expression in hyperplastic prostates of *PSA-Cre;Pten-loxP/loxP* mice, whereas the expression of Sca-1, CD44 and CD117 mRNA was increased compared to the normal prostate (H. Korsten, unpublished). These data indicate that the CD49f^+^ and the Sca-1^+^CD133^+^CD44^+^CD117^+^ cells are different from the lineage-specific luminal progenitor cells identified in this study (Figure 7). Our preliminary findings can form the basis for further isolation and functional characterization of hyperplastic cells from prostates of *PSA-Cre;Pten-loxP/loxP* mice, and of luminal epithelial progenitor cells from the more distal region of the normal prostate.

The proximal region of the prostate has been proposed as a stem/progenitor cell niche [28]–[30]. As shown here, the more compact Sca-1^+^ cells in the luminal epithelial cell layer in the proximal prostate express many markers of hyperplastic prostate cells and of progenitors of luminal epithelial cells in the more distal prostate (Figure 5 and 6). High expression of *integrinα2 (CD49b)* and *CD44* was observed in the proximal prostate (H. Korsten, unpublished) [30].

Despite a similar gene expression profile, hyperplasia in *Pten* knockout mice did not develop from luminal epithelial cells in the proximal prostate. In contrast to the distal cells, we did not observe pAkt overexpression in the proximal cells, suggesting that *Pten* is not inactivated in the proximal prostate. At present, it is unknown whether this is due to low Cre expression or limited susceptibility of the *Pten* locus to recombination in this specific part of the prostate. Alternatively, *Pten* is inactivated, but PI3K/PTEN signaling in these cells is not induced. It should be noted that in *PB-Cre;Trp53/Rb* knockout mice tumors arise from the proximal prostate, indicating that proximal cells can function as tumor initiating cells in cancer models [32].

Recently, fusions between genes encoding ETS transcription factors and genes encoding prostate-specific genes, mostly *TMPRSS2-ERG*, have been reported as most frequent genetic alteration in early stages of human prostate cancer [58]. Interestingly, *Tmprss2* expression is induced late during mouse prostate development [59]. We also found a fusion between the *KLK2* gene and the ETS gene *ETV4* in clinical prostate cancer [60]. *KLK2* is highly homologous to the *PSA (KLK3)* gene used in our mouse prostate cancer model for *Cre* expression. Although it is unknown in which cell type the gene fusions occur, because of the high prostate-specificity of most genes that are coupled to ETS genes it is tempting to speculate that the genetic alterations occur and/or become manifest in progenitors of luminal epithelial cells and not in tissue stem cells or multi-potent progenitor cells. These findings clearly indicate the high importance of identification of progenitor cells of luminal epithelial cells in the human prostate and further characterization of the mouse progenitors.

## Materials and Methods

### Generation of Prostate Targeted Pten Knockout Mice

The generation of *PSA-Cre* mice (strain FVB), mice carrying the *Pten-loxP* allele (strain 129Ola), and bi-allelic and mono-allelic prostate *Pten* knockout mice have been described previously [13], [61]. Mice were housed according to guidelines of the Erasmus Medical Center, and procedures were carried out in compliance with standards for use of laboratory animals. Animal experiments performed in this manuscript have been approved by the animal experimental committee of the Erasmus Medical Center (DEC-consult).

### RNA extraction, cDNA preparation and QPCR analysis

RNA was isolated from frozen mouse prostates using the Qiagen RNeasy RNA extraction Kit (Qiagen, Hilden, Germany) according to the manufacturer's guidelines, including an on column DNAseI digestion. RNA quality was checked by agarose gel electrophoresis. For RNA extraction from normal and hyperplastic prostates at 2m and 4–5m a pool of prostate lobes of one mouse was used. Pools of prostates from five mice were used for each RNA sample from proximal and distal prostate regions of control littermates (4–5m).

The methods for cDNA preparation and QPCR analysis were described previously [60]. Primer sequences are given in Table S3. The expression level of target genes was determined relative to the endogenous reference *Hypoxanthine-guanine Phosphoribosyltransferase (Hprt).*

### Immunohistochemistry and Immunofluorescent double staining

Tissues were fixed in buffered 4% formalin for ∼16h at room temperature, dehydrated, embedded in paraffin and sections were cut at 4 µm. Antibodies used for immunohistochemistry and immunofluorescence are listed in Table S4. The Nkx3.1 antibody was a kind gift from Dr. Cory Abate-Shen. Microwave treatment was applied for antigen retrieval by boiling in 10 mM sodium citrate (pH 6.0) for 15 min. For CK19 staining, tissue sections were pepsine (0.5%) treated for 30 min at 37°C. Primary antibodies incubation was overnight at 4°C. For immunohistochemistry, tissue sections were incubated with biotin labeled secondary antibody for 1h at room temperature. Immunoreactivity was visualized by streptavidin-peroxidase incubation (HK320-UK, 1∶50, BioGenex, San Ramon, CA). For immunofluorescent pAkt, CK8 and p63 staining, tissue sections were incubated with FITC/TRITC labeled secondary antibodies (Table S4). The signals for Clu and Tacstd2 were visualized by incubation with Rabbit Anti-Goat biotin followed by Streptavidin-TRITC. Anti-fading fluorescent mounting medium (H-1000, Vector laboratories, Burlingame, CA) containing DAPI (1∶2000, Sigma Chemical, St.Louis, MO) was used to cover the slide. Immunofluorescent stained slides were analyzed with a DMRXA microscope (Leica, Wetzlar, Germany).

To estimate the number of progenitor cells in the luminal epithelial cell layer of normal prostates at 4–5w and at 4–5m, slices of the mouse anterior lobe were stained for Clu. By counting the total cell number and the number of Clu^+^ cells in a prostate lobe, an estimation of the frequency of Clu^+^ cells could be made.

### cDNA microarray hybridization and analysis

The cDNA microarrays were hybridized and normalized as described previously [62], [63]. The common reference, a mixture of RNAs isolated from mouse adult testis and prostate, was in all experiments Cy5-labeled. The cDNA microarrays were manufactured at the Central Microarray Facility at the Netherlands Cancer Institute (NKI, Amsterdam, The Netherlands) and contained 16,416 spots.

Principal component analysis was performed using the Spotfire software package (Spotfire, Inc., Sommerville, MA; spotfire decision site V8.1). For unsupervised hierarchical clustering and visualization of differentially expressed genes the programs Cluster and Treeview were used [64]. After setting criteria for unsupervised hierarchical clustering of normal and hyperplastic prostates (100% signal and at least 2 observations of |0.8|) 528 genes were selected. Within Treeview, the image contrast and mask value settings were 1.5 and 0.2 respectively for all figures.

The difference in mean mRNA expression level of a gene was calculated by substracting the log2 transformed average expression level in normal prostates from the average expression level in hyperplastic prostates. Genes were ranked based on gene expression level difference. Figure 1 shows the mean expression level differences of known genes, which gave a signal in at least two experiments in one group. In addition, by performing Significance Analysis of Microarrays (SAM) (Version 1.21) [65] gene expression levels differences relative to the standard deviation of these expression levels within one group was calculated. By SAM genes with an at least 2 fold change in ratio were identified. The q-value (false discovery rate) for genes identified by SAM analysis was 0.15%.

Microarray data have been submitted to the ArrayExpress public database (Accession E-MEXP-2029).

## Supporting Information

Figure S1 Hyperplastic prostate cells of PSA-Cre;Pten-loxP/loxP mice have an epithelial progenitor cell phenotype. (A) QPCR analysis of epithelial cell marker expression in hyperplastic prostates at 2m and 4–5m. Each group was compased of five RNA samples. Data are shown as average expression levels +/− SE relative to Hprt expression. NP: normal prostate, HP: hyperplastic prostate. (B–I) Immunohistochemical analysis of epithelial cell markers in the mouse anterior lobe of normal and hyperplastic prostates at 4–5m. (B) Nkx3.1 NP, (C) Nkx3.1 HP, (D) CK8 NP, (E) CK8 HP, (F) CK19 NP, (G) CK19 HP, (H) P63 NP and (I) P63 HP.
(8.51 MB TIF)

Figure S2 QPCR analysis of the five genes with the lowest expression in hyperplastic prostates (4–5m). Each group was composed of five RNA samples. The expression levels in 2m and 4–5m old hyperplastic prostates are shown as average expression level +/− SE relative to Hprt expression.
(6.62 MB TIF)

Figure S3 Number of Clu+ hyperplastic foci in prostates of PSA-Cre;Pten-loxP/+ mice. At older age (>11m) the number of Clu+ hyperplastic foci increased in prostates of PSA-Cre;Pten-loxP/+ mice. Clu+ hyperplastic foci were counted in fifteen consecutive slides of a longitudinal embedded anterior prostate lobe. At each time point the prostates of three mice were analyzed.
(8.78 MB TIF)

Figure S4 Tacstd2+ cells in the P63+ basal epithelial cell layer of a normal mouse prostate (4–5w).
(6.49 MB TIF)

Figure S5 Immunohistochemical analysis of epithelial cell markers and hyperplastic cell markers in urethra of wild type mice. (A) CK8, (B) P63, (C) Ppp1r1b, (D) Tacstd2, (E) Clu and (F) Sca-1.
(6.95 MB TIF)

Table S1 Full gene names of genes overexpressed in hyperplastic prostates of PSA-Cre;Pten-loxP/loxP mice.
(0.03 MB DOC)

Table S2 Genes with significantly differentially expression in hyperplastic prostates of PSA-Cre;Pten-loxP/loxP mice as determined by SAM.
(0.19 MB DOC)

Table S3 Primer sequences of genes analyzed by QPCR.
(0.03 MB DOC)

Table S4 Information of antibodies used for immunohistochemistry and immunofluorescence
(0.03 MB DOC)

## References

[1] Jemal A, Siegel R, Ward E, Hao Y, Xu J (2008). Cancer statistics, 2008.. CA Cancer J Clin.

[2] Majumder PK, Sellers WR (2005). Akt-regulated pathways in prostate cancer.. Oncogene.

[3] Salmena L, Carracedo A, Pandolfi PP (2008). Tenets of PTEN tumor suppression.. Cell.

[4] Leslie NR, Downes CP (2004). PTEN function: how normal cells control it and tumour cells lose it.. Biochem J.

[5] Hamada K, Sasaki T, Koni PA, Natsui M, Kishimoto H (2005). The PTEN/PI3K pathway governs normal vascular development and tumor angiogenesis.. Genes Dev.

[6] Stiles B, Groszer M, Wang S, Jiao J, Wu H (2004). PTENless means more.. Dev Biol.

[7] Shen WH, Balajee AS, Wang J, Wu H, Eng C (2007). Essential role for nuclear PTEN in maintaining chromosomal integrity.. Cell.

[8] Akala OO, Clarke MF (2006). Hematopoietic stem cell self-renewal.. Curr Opin Genet Dev.

[9] Rossi DJ, Weissman IL (2006). Pten, tumorigenesis, and stem cell self-renewal.. Cell.

[10] Di Cristofano A, Pesce B, Cordon-Cardo C, Pandolfi PP (1998). Pten is essential for embryonic development and tumour suppression.. Nat Genet.

[11] Suzuki A, de la Pompa JL, Stambolic V, Elia AJ, Sasaki T (1998). High cancer susceptibility and embryonic lethality associated with mutation of the PTEN tumor suppressor gene in mice.. Curr Biol.

[12] Backman SA, Ghazarian D, So K, Sanchez O, Wagner KU (2004). Early onset of neoplasia in the prostate and skin of mice with tissue-specific deletion of Pten.. Proc Natl Acad Sci U S A.

[13] Ma X, Ziel-van der Made AC, Autar B, van der Korput HA, Vermeij M (2005). Targeted biallelic inactivation of Pten in the mouse prostate leads to prostate cancer accompanied by increased epithelial cell proliferation but not by reduced apoptosis.. Cancer Res.

[14] Trotman LC, Niki M, Dotan ZA, Koutcher JA, Di Cristofano A (2003). Pten dose dictates cancer progression in the prostate.. PLoS Biol.

[15] Wang S, Gao J, Lei Q, Rozengurt N, Pritchard C (2003). Prostate-specific deletion of the murine Pten tumor suppressor gene leads to metastatic prostate cancer.. Cancer Cell.

[16] Rossi DJ, Jamieson CH, Weissman IL (2008). Stems cells and the pathways to aging and cancer.. Cell.

[17] Orkin SH, Zon LI (2008). Hematopoiesis: an evolving paradigm for stem cell biology.. Cell.

[18] Wang JC, Dick JE (2005). Cancer stem cells: lessons from leukemia.. Trends Cell Biol.

[19] Ailles LE, Weissman IL (2007). Cancer stem cells in solid tumors.. Curr Opin Biotechnol.

[20] Reya T, Morrison SJ, Clarke MF, Weissman IL (2001). Stem cells, cancer, and cancer stem cells.. Nature.

[21] Lam JS, Reiter RE (2006). Stem cells in prostate and prostate cancer development.. Urol Oncol.

[22] Campbell LL, Polyak K (2007). Breast tumor heterogeneity: cancer stem cells or clonal evolution?. Cell Cycle.

[23] Adams JM, Strasser A (2008). Is tumor growth sustained by rare cancer stem cells or dominant clones?. Cancer Res.

[24] Visvader JE, Lindeman GJ (2008). Cancer stem cells in solid tumours: accumulating evidence and unresolved questions.. Nat Rev Cancer.

[25] English HF, Santen RJ, Isaacs JT (1987). Response of glandular versus basal rat ventral prostatic epithelial cells to androgen withdrawal and replacement.. Prostate.

[26] Collins AT, Maitland NJ (2006). Prostate cancer stem cells.. Eur J Cancer.

[27] Lawson DA, Witte ON (2007). Stem cells in prostate cancer initiation and progression.. J Clin Invest.

[28] Burger PE, Xiong X, Coetzee S, Salm SN, Moscatelli D (2005). Sca-1 expression identifies stem cells in the proximal region of prostatic ducts with high capacity to reconstitute prostatic tissue.. Proc Natl Acad Sci U S A.

[29] Tsujimura A, Koikawa Y, Salm S, Takao T, Coetzee S (2002). Proximal location of mouse prostate epithelial stem cells: a model of prostatic homeostasis.. J Cell Biol.

[30] Leong KG, Wang BE, Johnson L, Gao WQ (2008). Generation of a prostate from a single adult stem cell.. Nature.

[31] Wang S, Garcia AJ, Wu M, Lawson DA, Witte ON (2006). Pten deletion leads to the expansion of a prostatic stem/progenitor cell subpopulation and tumor initiation.. Proc Natl Acad Sci U S A.

[32] Zhou Z, Flesken-Nikitin A, Nikitin AY (2007). Prostate cancer associated with p53 and Rb deficiency arises from the stem/progenitor cell-enriched proximal region of prostatic ducts.. Cancer Res.

[33] Rizzo S, Attard G, Hudson DL (2005). Prostate epithelial stem cells.. Cell Prolif.

[34] Litvinov IV, De Marzo AM, Isaacs JT (2003). Is the Achilles' heel for prostate cancer therapy a gain of function in androgen receptor signaling?. J Clin Endocrinol Metab.

[35] Hudson DL, Guy AT, Fry P, O'Hare MJ, Watt FM (2001). Epithelial cell differentiation pathways in the human prostate: identification of intermediate phenotypes by keratin expression.. J Histochem Cytochem.

[36] Wang Y, Hayward S, Cao M, Thayer K, Cunha G (2001). Cell differentiation lineage in the prostate.. Differentiation.

[37] Fornaro M, Dell'Arciprete R, Stella M, Bucci C, Nutini M (1995). Cloning of the gene encoding Trop-2, a cell-surface glycoprotein expressed by human carcinomas.. Int J Cancer.

[38] Goldstein AS, Lawson DA, Cheng D, Sun W, Garraway IP (2008). Trop2 identifies a subpopulation of murine and human prostate basal cells with stem cell characteristics.. Proc Natl Acad Sci U S A.

[39] Bonkhoff H, Remberger K (1996). Differentiation pathways and histogenetic aspects of normal and abnormal prostatic growth: a stem cell model.. Prostate.

[40] Backman S, Stambolic V, Mak T (2002). PTEN function in mammalian cell size regulation.. Curr Opin Neurobiol.

[41] Ellwood-Yen K, Graeber TG, Wongvipat J, Iruela-Arispe ML, Zhang J (2003). Myc-driven murine prostate cancer shares molecular features with human prostate tumors.. Cancer Cell.

[42] Greenberg NM, DeMayo F, Finegold MJ, Medina D, Tilley WD (1995). Prostate cancer in a transgenic mouse.. Proc Natl Acad Sci U S A.

[43] Han G, Buchanan G, Ittmann M, Harris JM, Yu X (2005). Mutation of the androgen receptor causes oncogenic transformation of the prostate.. Proc Natl Acad Sci U S A.

[44] Kim MJ, Bhatia-Gaur R, Banach-Petrosky WA, Desai N, Wang Y (2002). Nkx3.1 mutant mice recapitulate early stages of prostate carcinogenesis.. Cancer Res.

[45] Maddison LA, Sutherland BW, Barrios RJ, Greenberg NM (2004). Conditional deletion of Rb causes early stage prostate cancer.. Cancer Res.

[46] Acevedo VD, Gangula RD, Freeman KW, Li R, Zhang Y (2007). Inducible FGFR-1 activation leads to irreversible prostate adenocarcinoma and an epithelial-to-mesenchymal transition.. Cancer Cell.

[47] Cleutjens KB, van der Korput HA, Ehren-van Eekelen CC, Sikes RA, Fasciana C (1997). A 6-kb promoter fragment mimics in transgenic mice the prostate-specific and androgen-regulated expression of the endogenous prostate-specific antigen gene in humans.. Mol Endocrinol.

[48] Groszer M, Erickson R, Scripture-Adams DD, Dougherty JD, Le Belle J (2006). PTEN negatively regulates neural stem cell self-renewal by modulating G0-G1 cell cycle entry.. Proc Natl Acad Sci U S A.

[49] Groszer M, Erickson R, Scripture-Adams DD, Lesche R, Trumpp A (2001). Negative regulation of neural stem/progenitor cell proliferation by the Pten tumor suppressor gene in vivo.. Science.

[50] Liu X, Bruxvoort KJ, Zylstra CR, Liu J, Cichowski R (2007). Lifelong accumulation of bone in mice lacking Pten in osteoblasts.. Proc Natl Acad Sci U S A.

[51] Modur V, Nagarajan R, Evers BM, Milbrandt J (2002). FOXO proteins regulate tumor necrosis factor-related apoptosis inducing ligand expression. Implications for PTEN mutation in prostate cancer.. J Biol Chem.

[52] Parsons JK, Gage WR, Nelson WG, De Marzo AM (2001). p63 protein expression is rare in prostate adenocarcinoma: implications for cancer diagnosis and carcinogenesis.. Urology.

[53] Xin L, Lawson DA, Witte ON (2005). The Sca-1 cell surface marker enriches for a prostate-regenerating cell subpopulation that can initiate prostate tumorigenesis.. Proc Natl Acad Sci U S A.

[54] Xin L, Lukacs RU, Lawson DA, Cheng D, Witte ON (2007). Self-renewal and multilineage differentiation in vitro from murine prostate stem cells.. Stem Cells.

[55] Lawson DA, Xin L, Lukacs RU, Cheng D, Witte ON (2007). Isolation and functional characterization of murine prostate stem cells.. Proc Natl Acad Sci U S A.

[56] Collins AT, Berry PA, Hyde C, Stower MJ, Maitland NJ (2005). Prospective identification of tumorigenic prostate cancer stem cells.. Cancer Res.

[57] Patrawala L, Calhoun-Davis T, Schneider-Broussard R, Tang DG (2007). Hierarchical organization of prostate cancer cells in xenograft tumors: the CD44+alpha2beta1+ cell population is enriched in tumor-initiating cells.. Cancer Res.

[58] Kumar-Sinha C, Tomlins SA, Chinnaiyan AM (2008). Recurrent gene fusions in prostate cancer.. Nat Rev Cancer.

[59] Hermans KG, van der Korput HA, van Marion R, van de Wijngaart DJ, Ziel-van der Made A (2008). Truncated ETV1, fused to novel tissue-specific genes, and full-length ETV1 in prostate cancer.. Cancer Res.

[60] Hermans KG, Bressers AA, van der Korput HA, Dits NF, Jenster G (2008). Two unique novel prostate-specific and androgen-regulated fusion partners of ETV4 in prostate cancer.. Cancer Res.

[61] Marino S, Krimpenfort P, Leung C, van der Korput HA, Trapman J (2002). PTEN is essential for cell migration but not for fate determination and tumourigenesis in the cerebellum.. Development.

[62] Baugh LR, Hill AA, Brown EL, Hunter CP (2001). Quantitative analysis of mRNA amplification by in vitro transcription.. Nucleic Acids Res.

[63] Hendriksen PJ, Dits NF, Kokame K, Veldhoven A, van Weerden WM (2006). Evolution of the androgen receptor pathway during progression of prostate cancer.. Cancer Res.

[64] Eisen MB, Spellman PT, Brown PO, Botstein D (1998). Cluster analysis and display of genome-wide expression patterns.. Proc Natl Acad Sci U S A.

[65] Tusher VG, Tibshirani R, Chu G (2001). Significance analysis of microarrays applied to the ionizing radiation response.. Proc Natl Acad Sci U S A.

