# Supplementary figures and images for: Accumulating Progenitor Cells in the Luminal Epithelial Cell Layer Are Candidate Tumor Initiating Cells in a Pten Knockout Mouse Prostate Cancer Model
Source: PLoS One. 2009 May 22;4(5):e5662. doi: 10.1371/journal.pone.0005662 (PMC2680948; doi:10.1371/journal.pone.0005662)

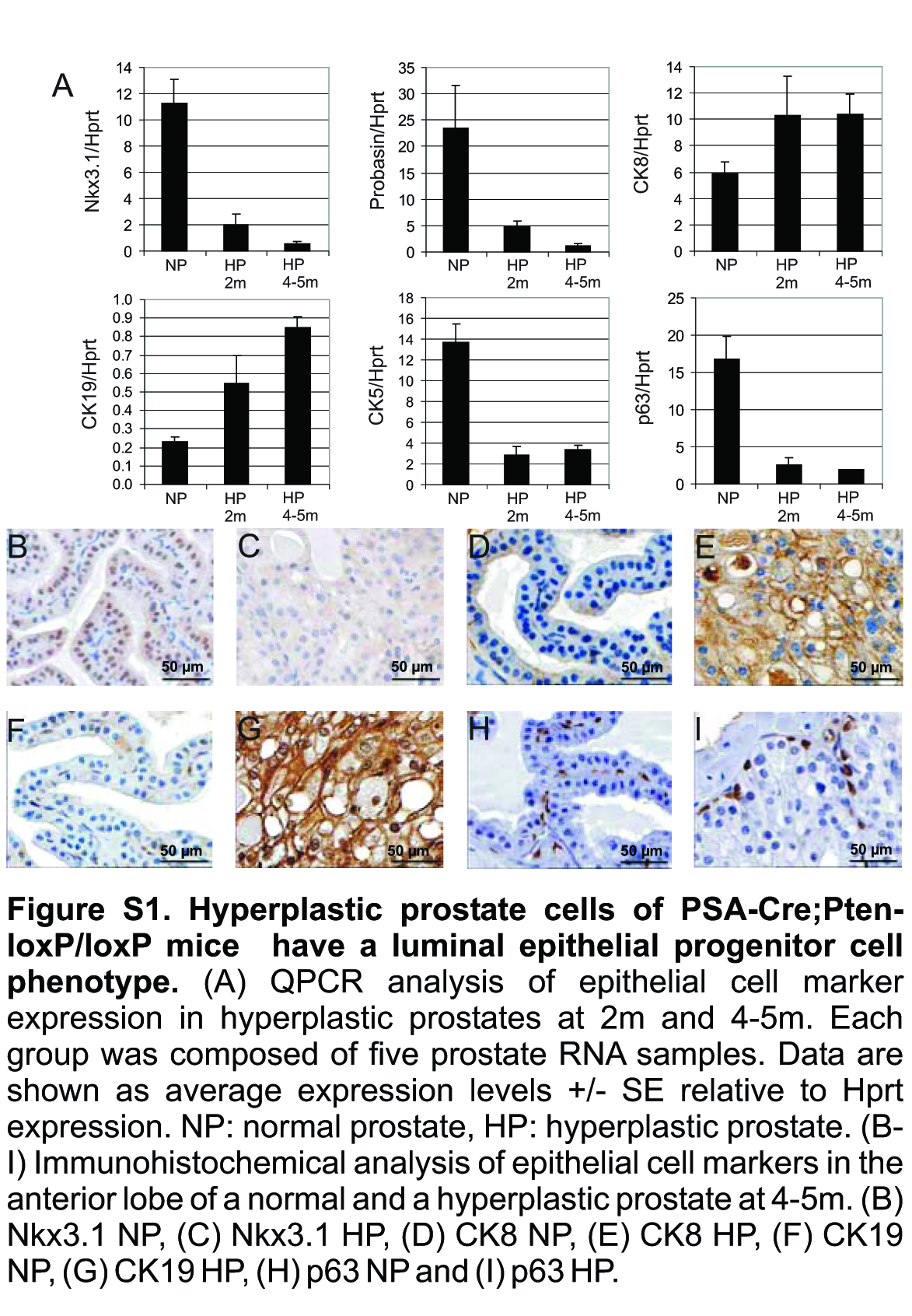

Supplement: Figure S1 — Hyperplastic prostate cells of PSA-Cre;Pten-loxP/loxP mice have an epithelial progenitor cell phenotype. (A) QPCR analysis of epithelial cell marker expression in hyperplastic prostates at 2m and 4–5m. Each group was compased of five RNA samples. Data are shown as average expression levels +/− SE relative to Hprt expression. NP: normal prostate, HP: hyperplastic prostate. (B–I) Immunohistochemical analysis of epithelial cell markers in the mouse anterior lobe of normal and hyperplastic prostates at 4–5m. (B) Nkx3.1 NP, (C) Nkx3.1 HP, (D) CK8 NP, (E) CK8 HP, (F) CK19 NP, (G) CK19 HP, (H) P63 NP and (I) P63 HP. (8.51 MB TIF) [file pone.0005662.s001.tif]

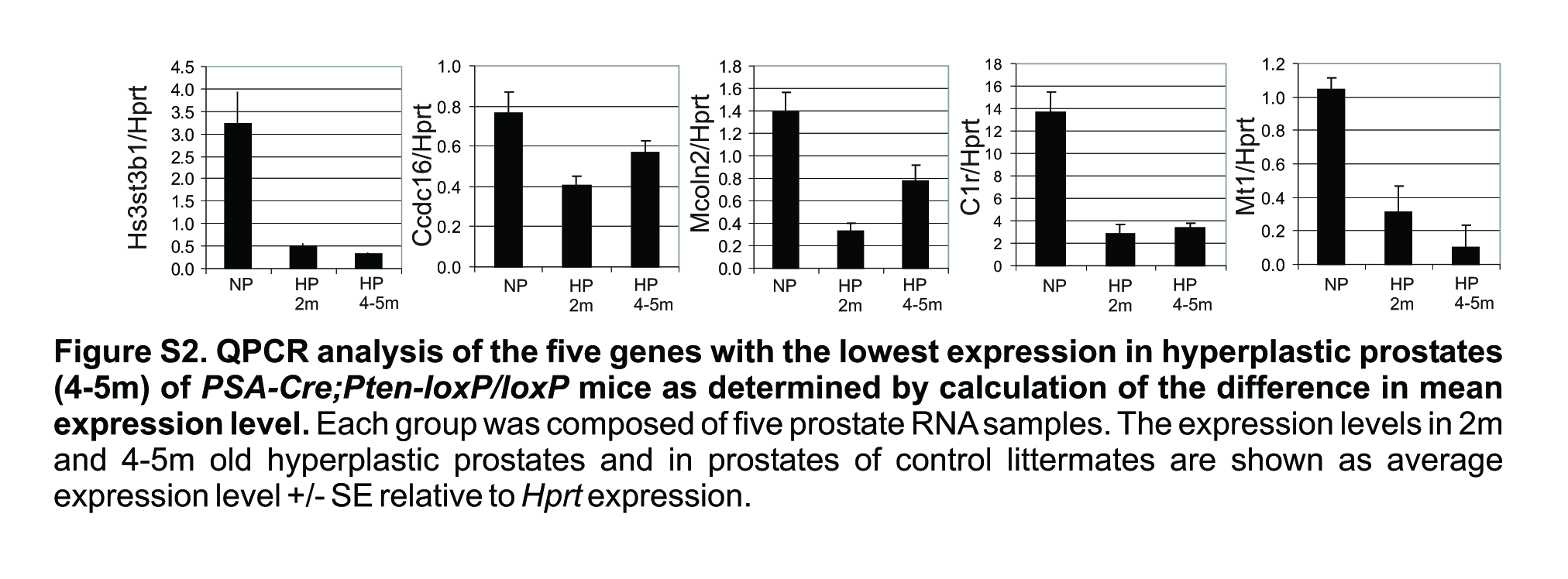

Supplement: Figure S2 — QPCR analysis of the five genes with the lowest expression in hyperplastic prostates (4–5m). Each group was composed of five RNA samples. The expression levels in 2m and 4–5m old hyperplastic prostates are shown as average expression level +/− SE relative to Hprt expression. (6.62 MB TIF) [file pone.0005662.s002.tif]

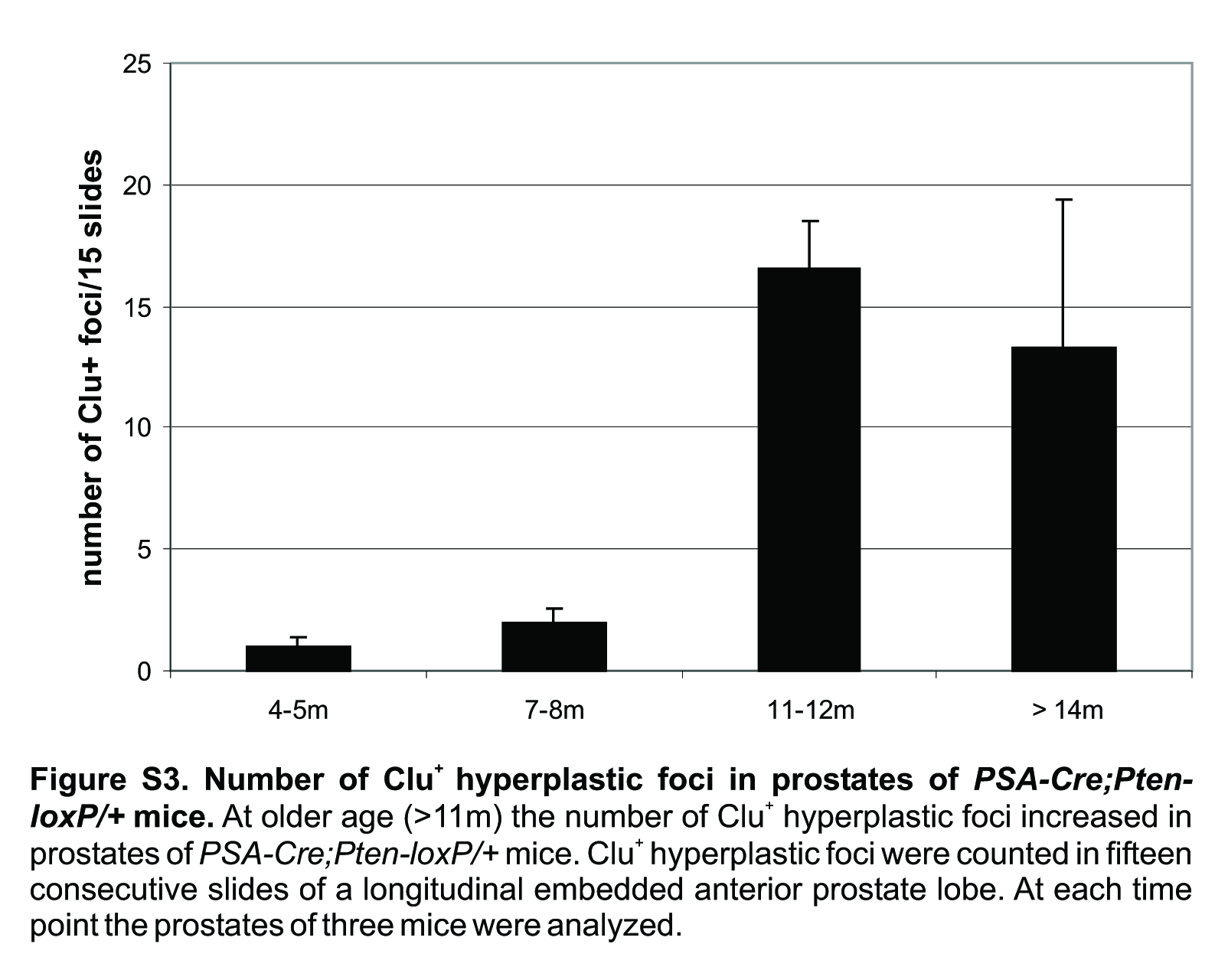

Supplement: Figure S3 — Number of Clu+ hyperplastic foci in prostates of PSA-Cre;Pten-loxP/+ mice. At older age (>11m) the number of Clu+ hyperplastic foci increased in prostates of PSA-Cre;Pten-loxP/+ mice. Clu+ hyperplastic foci were counted in fifteen consecutive slides of a longitudinal embedded anterior prostate lobe. At each time point the prostates of three mice were analyzed. (8.78 MB TIF) [file pone.0005662.s003.tif]

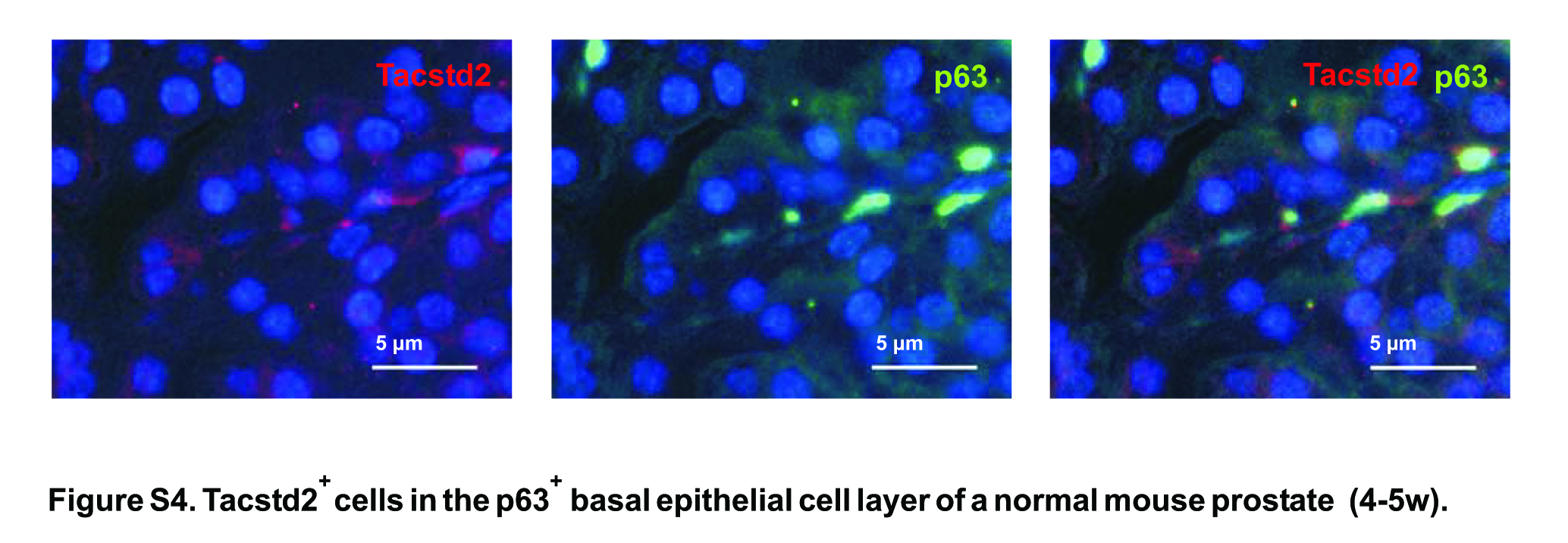

Supplement: Figure S4 — Tacstd2+ cells in the P63+ basal epithelial cell layer of a normal mouse prostate (4–5w). (6.49 MB TIF) [file pone.0005662.s004.tif]

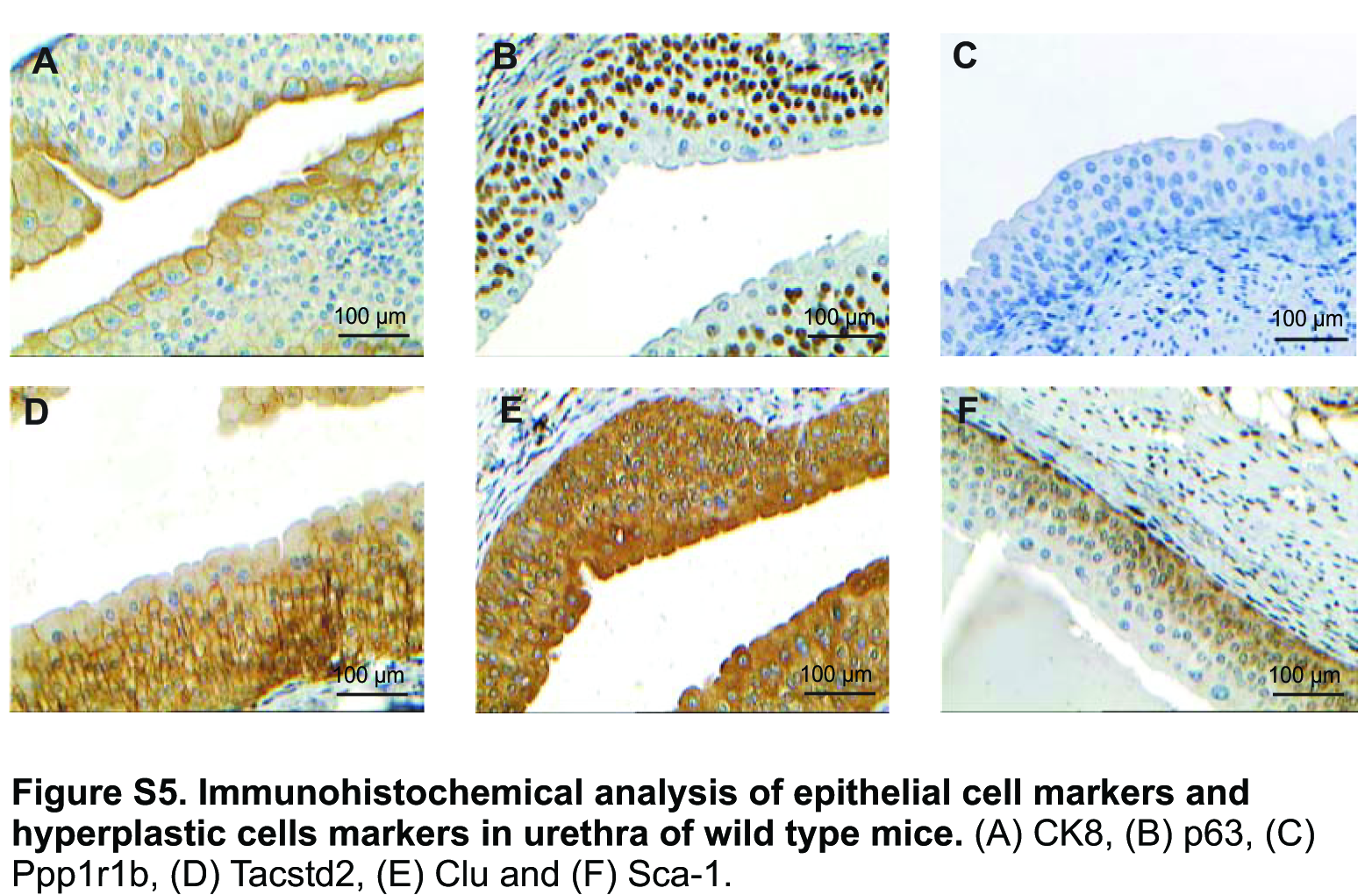

Supplement: Figure S5 — Immunohistochemical analysis of epithelial cell markers and hyperplastic cell markers in urethra of wild type mice. (A) CK8, (B) P63, (C) Ppp1r1b, (D) Tacstd2, (E) Clu and (F) Sca-1. (6.95 MB TIF) [file pone.0005662.s005.tif]
