# Supplementary material for: Accumulating Progenitor Cells in the Luminal Epithelial Cell Layer Are Candidate Tumor Initiating Cells in a Pten Knockout Mouse Prostate Cancer Model
Source: PLoS One. 2009 May 22;4(5):e5662. doi: 10.1371/journal.pone.0005662 (PMC2680948; doi:10.1371/journal.pone.0005662)
Supplement: Table S1 — Full gene names of genes overexpressed in hyperplastic prostates of PSA-Cre;Pten-loxP/loxP mice. (0.03 MB DOC) [file pone.0005662.s006.doc]

**Table S1. Full names of genes overexpressed in hyperplastic prostates of *PSA-Cre;Pten-loxP/loxP* mice.**

| Abbreviation | Gene Name |
| --- | --- |
| **Expi** | Extracellular proteinase inhibitor |
| **Wfdc2** | WAP four-disulfide core domain 2 |
| **Tacstd2** | Tumour-associated calcium signal transducer 2 |
| **Clu** | Clusterin |
| **Ppp1r1b** | Protein phosphatase 1, regulatory (inhibitor) subunit 1B |
| **Lcn2** | Lipocalin 2 |
| **Slc39a4** | Solute carrier family 39 (zinc transporter), member 4 |
| **Zfand2b** | Zinc finger, AN1 type domain2B |
| **Ckmt1** | Creatine kinase, mitochondrial 1, ubiquitous |
| **Anxa3** | Annexin 3 |
| **Mlp** | Marcks-like protein |
| **Otud1** | OUT domain containing 1 |
| **Klf5** | Kruppel-like factor 5 |
| **Ly6a** | Lymphocyte antigen 6 complex, locus A |
| **Cbr2** | Carbonyl reductase 2 |
| **Scotin** | Scotin |
| **Slc12a2** | Solute carrier family 12, member 2 |
| **Pank3** | Pantothenate kinase 3 |
| **Nupr1** | Nuclear protein 1 |
| **Ptpn21** | Protein tyrosine phosphatase, non-receptor type 21 |
