# Supplementary material for: Accumulating Progenitor Cells in the Luminal Epithelial Cell Layer Are Candidate Tumor Initiating Cells in a Pten Knockout Mouse Prostate Cancer Model
Source: PLoS One. 2009 May 22;4(5):e5662. doi: 10.1371/journal.pone.0005662 (PMC2680948; doi:10.1371/journal.pone.0005662)
Supplement: Table S2 — Genes with significantly differentially expression in hyperplastic prostates of PSA-Cre;Pten-loxP/loxP mice as determined by SAM. (0.19 MB DOC) [file pone.0005662.s007.doc]

**Table S2. Significantly differentially expressed genes in hyperplastic prostates of *PSA-Cre;Pten-loxP/loxP* mice as assayed by SAM analysis.**

## Significant upregulated genes

| **Accesion Code** | **Unigene Number** | **Gene Name** | **Score(d)** | **Fold Change** |
| --- | --- | --- | --- | --- |
| BG071358 | Mm.1650 | Expi | 11.47 | 22,35956 |
| BG071688 | Mm.250165 | Mamdc1 | 10.57 | 8,98338 |
| BG066797 | Mm.154045 | Tacstd2 | 8.77 | 8,23533 |
| BG072238 | Mm.27289 | Wfdc2 | 8.58 | 10,00670 |
| BG065849 | Mm.5574 | Slc22a19 | 7.41 | 5,48686 |
| BG071114 | Mm.20675 | Otud1 | 7.02 | 5,50665 |
| BG070865 | Mm.259645 | Pank3 | 6.96 | 4,69510 |
| BG072793 | Mm.8180 | Sca-1 | 6.91 | 5,19812 |
| BG064167 | Mm.2769 | Mlp | 6.46 | 5,54302 |
| BG070413 | Mm.7612 | Ltf | 6.41 | 3,49584 |
| BG065106 | Mm.2769 | Mlp | 6.38 | 5,46968 |
| BG071309 | Mm.28497 | Ppp1r1b | 6.25 | 8,09515 |
| BG069030 | Mm.4420 | Ptpn21 | 6.23 | 4,40579 |
| BG066823 | Mm.970 | Ckmt1 | 6.19 | 6,15851 |
| BG064706 | Mm.1012 | CK19 | 6.15 | 3,65135 |
| BG076188 | Mm.29483 | Slc39a4 | 6.00 | 6,18341 |
| BG069863 | Mm.154660 | Plat | 5.99 | 3,80224 |
| BG065118 | Mm.90587 | Eno1 | 5.68 | 3,90298 |
| BG070839 | Mm.7214 | Anxa3 | 5.45 | 5,33097 |
| BG069367 | Mm.32646 | Zfand2b | 5.25 | 6,17232 |
| BG074576 | Mm.4168 | Slc12a2 | 5.12 | 4,41512 |
| BG074344 | Mm.17510 | Msln | 5.03 | 11,64736 |
| BG065116 | Mm.90587 | Eno1 | 5.01 | 3,25239 |
| BG071514 | Mm.235090 | Tcfcp2l1 | 4.97 | 3,20175 |
| BG072403 | Mm.250158 | Rnf11 | 4.89 | 3,77734 |
| BG067864 | Mm.29587 | LOC227616 | 4.87 | 3,63909 |
| BG076074 | Mm.18814 | Baiap2l1 | 4.86 | 3,64365 |
| BG071705 | Mm.30262 | Klf5 | 4.76 | 5,24630 |
| BG074971 | Mm.24510 | Slc12a7 | 4.74 | 2,86031 |
| BG068045 | Mm.38441 | Galnt3 | 4.66 | 2,78278 |
| BG067918 | Mm.903 | Btg2 | 4.62 | 4,18896 |
| BG067532 | Mm.1662 | Fxyd3 | 4.60 | 2,82506 |
| BG064389 | Mm.30010 | Arpc1b | 4.60 | 2,69594 |
| BG074458 | Mm.234242 | Dhcr24 | 4.57 | 3,24006 |
| BG064659 | Mm.23575 | Gprc5a | 4.52 | 3,17411 |
| BG067693 | Mm.25613 | Ier3 | 4.47 | 2,94791 |
| BG075912 | Mm.206505 | Timp2 | 4.47 | 3,13271 |
| BG071245 | Mm.21454 | Cbr2 | 4.38 | 4,97431 |
| BG068068 | Mm.27365 | Fat1 | 4.37 | 2,34774 |
| BG067727 | Mm.28518 | Tnfrsf12a | 4.37 | 3,73157 |
| BG073464 | Mm.28986 | Sqrdl | 4.35 | 2,46512 |
| BG075943 | Mm.219635 | Ptplad1 | 4.34 | 3,28708 |
| BG063173 | Mm.23961 | Actn1 | 4.33 | 2,76328 |
| BG067375 | Mm.23939 | Tpo1 | 4.21 | 2,30059 |
| BG074770 | Mm.195800 | Rgnef | 4.19 | 2,76110 |
| BG071322 | Mm.21454 | Cbr2 | 4.18 | 3,85257 |
| BG069628 | Mm.12246 | Ier5 | 4.14 | 3,10972 |
| BG064378 | Mm.24745 | Bzw2 | 4.11 | 2,44005 |
| BG072100 | Mm.9772 | Chdh | 4.10 | 2,69430 |
| BG071742 | Mm.22547 | Asah1 | 4.10 | 2,11695 |
| BG075635 | Mm.29619 | Gga2 | 4.09 | 2,10064 |
| BG070491 | Mm.100144 | S100a6 | 4.08 | 2,19837 |
| BG069386 | Mm.11662 | Slco4c1 | 4.07 | 2,34379 |
| BG069616 | Mm.70573 | Pde2a | 4.04 | 2,29157 |
| BG065926 | Mm.35523 | Adnp | 4.03 | 2,64795 |
| BG069516 | Mm.41401 | Slc6a8 | 4.02 | 2,24963 |
| BG071387 | Mm.30239 | Slc39a8 | 4.01 | 4,46718 |
| BG065159 | Mm.200936 | Sh2b3 | 3.98 | 2,38959 |
| BG075302 | Mm.135092 | Slc4a7 | 3.96 | 3,04537 |
| BG064186 | Mm.28954 | Arhu | 3.95 | 2,44913 |
| BG069782 | Mm.4426 | Cd63 | 3.94 | 2,10084 |
| BG071171 | Mm.282 | Gsto1 | 3.93 | 3,45906 |
| BG072122 | Mm.18814 | Baiap2l1 | 3.93 | 3,08255 |
| BG072291 | Mm.205601 | Cttn | 3.92 | 2,15382 |
| BG064085 | Mm.3963 | Elf3 | 3.92 | 2,66938 |
| BG063081 | Mm.3532 | Tmsb10 | 3.91 | 4,27950 |
| BG072110 | Mm.18742 | Nupr1 | 3.90 | 3,96276 |
| BG066817 | Mm.154725 | Ppapdc1 | 3.89 | 4,23496 |
| BG065384 | Mm.30017 | Gabarapl2 | 3.88 | 2,36392 |
| BG065470 | Mm.101062 | Ctnnb1 | 3.85 | 2,40472 |
| BG063612 | Mm.21203 | Zfp265 | 3.84 | 2,52583 |
| BG064050 | Mm.6800 | CK8 | 3.81 | 2,74686 |
| BG065103 | Mm.788 | Sca-2 | 3.81 | 2,92747 |
| BG063271 | Mm.44552 | Txnrd1 | 3.79 | 2,04798 |
| BG073255 | Mm.28484 | Tspan3 | 3.79 | 2,31680 |
| BG073025 | Mm.222584 | Pygb | 3.77 | 2,47069 |
| BG071200 | Mm.95879 | Jarid1a | 3.76 | 2,31542 |
| BG069505 | Mm.4168 | Slc12a2 | 3.75 | 3,93525 |
| BG064087 | Mm.25161 | Sirt2 | 3.73 | 2,17960 |
| BG068640 | Mm.11935 | Fbxo34 | 3.72 | 2,44193 |
| BG064070 | Mm.183102 | Actr3 | 3.71 | 2,01388 |
| BG070640 | Mm.26223 | Hace1 | 3.71 | 2,41787 |
| BG070089 | Mm.4259 | Tacstd1 | 3.70 | 2,23965 |
| BG066212 | Mm.34497 | Ltb4dh | 3.69 | 2,94193 |
| BG074931 | Mm.4303 | Ezh2 | 3.66 | 2,26496 |
| BG071031 | Mm.4825 | Mmp7 | 3.66 | 2,67935 |
| BG072281 | Mm.7286 | Ctbp1 | 3.65 | 2,84219 |
| BG074474 | Mm.31672 | Cdk6 | 3.65 | 2,64506 |
| BG064176 | Mm.4554 | Lgals3 | 3.63 | 2,58095 |
| BG069726 | Mm.4168 | Slc12a2 | 3.63 | 3,16789 |
| BG073116 | Mm.29524 | Clic2 | 3.62 | 2,69005 |
| BG075859 | Mm.73682 | Tmepai | 3.61 | 2,66887 |
| BG072707 | Mm.2734 | Sat | 3.61 | 2,84646 |
| BG070245 | Mm.4168 | Slc12a2 | 3.61 | 3,58739 |
| BG063000 | Mm.30060 | Avpi1 | 3.59 | 2,56568 |
| BG074494 | Mm.22119 | Fcgr3 | 3.59 | 2,29876 |
| BG075383 | Mm.27790 | Sfrs2ip | 3.58 | 2,22605 |
| BG072209 | Mm.200608 | Clu | 3.57 | 5,76677 |
| BG074542 | Mm.196533 | Scotin | 3.55 | 5,28095 |
| BG072254 | Mm.1377 | Tgfbr2 | 3.55 | 2,47781 |
| BG072077 | Mm.22248 | Igfbp4 | 3.54 | 2,94279 |
| BG063426 | Mm.30142 | CK7 | 3.54 | 2,26024 |
| BG063109 | Mm.21117 | Itgb4 | 3.54 | 2,20503 |
| BG075666 | Mm.8155 | Tgif | 3.52 | 2,48134 |
| BG063729 | Mm.5289 | Gapd | 3.50 | 2,57073 |
| BG070289 | Mm.448 | Cyba | 3.50 | 4,07125 |
| BG069415 | Mm.1519 | Ald | 3.50 | 2,15556 |
| BG069211 | Mm.3204 | Fdft1 | 3.49 | 2,03582 |
| BG063090 | Mm.30108 | Actr2 | 3.49 | 2,17786 |
| BG070902 | Mm.18742 | Nupr1 | 3.47 | 3,44853 |
| BG074463 | Mm.38450 | Septin9 | 3.46 | 2,03440 |
| BG072109 | Mm.390590 | Cntnap3 | 3.46 | 2,64455 |
| BG070106 | Mm.9537 | Lcn2 | 3.45 | 6,09852 |
| BG064639 | Mm.259329 | Cd2ap | 3.45 | 2,31525 |
| BG072676 | Mm.24808 | Fxyd6 | 3.43 | 2,86438 |
| BG063567 | Mm.14860 | Anxa1 | 3.43 | 2,19141 |
| BG069483 | Mm.205010 | Adipor1 | 3.43 | 2,27557 |
| BG066697 | Mm.1775 | Hn1 | 3.41 | 2,44874 |
| BG074171 | Mm.133872 | Stfa1 | 3.41 | 2,43656 |
| BG074532 | Mm.2159 | Bnip3 | 3.39 | 2,22193 |
| BG065385 | Mm.22192 | Slc31a1 | 3.38 | 2,49869 |
| BG065686 | Mm.1359 | uPar | 3.37 | 2,12928 |
| BG067214 | Mm.34268 | Cdc42ep5 | 3.36 | 2,03345 |
| BG068207 | Mm.11827 | Comtd1 | 3.36 | 2,26367 |
| BG076240 | Mm.28814 | Casp6 | 3.34 | 2,16681 |
| BG065049 | Mm.2423 | Col2a1 | 3.34 | 2,98133 |
| BG073190 | Mm.2662 | Gsta4 | 3.33 | 3,34865 |
| BG064907 | Mm.35581 | RIKEN cDNA 2310009O17 gene | 3.31 | 2,49474 |
| BG071601 | Mm.26700 | Tmem16a | 3.30 | 2,07314 |
| BG075211 | Mm.381 | Adfp | 3.29 | 2,80754 |
| BG067123 | Mm.35605 | Cdh1 | 3.27 | 2,79853 |
| BG069891 | Mm.231266 | Sestd1 | 3.26 | 2,25431 |
| BG074704 | Mm.27764 | Rnf128 | 3.26 | 2,01757 |
| BG067911 | Mm.7775 | Gdpd1 | 3.26 | 2,47217 |
| BG064165 | Mm.1620 | Anxa5 | 3.26 | 2,02078 |
| BG067594 | Mm.18718 | Scml1 | 3.24 | 2,02643 |
| BG071713 | Mm.214958 | Srebf1 | 3.22 | 2,05412 |
| BG070656 | Mm.18789 | Sox4 | 3.21 | 2,04055 |
| BG067160 | Mm.2538 | Pld2 | 3.19 | 2,38822 |
| BG071672 | Mm.18941 | Chmp2b | 3.18 | 2,03898 |
| BG072807 | Mm.156583 | CARP | 3.17 | 3,37532 |
| BG070386 | Mm.218846 | Lbp | 3.16 | 4,73538 |
| BG067840 | Mm.33819 | Trpm7 | 3.16 | 2,71071 |
| BG075881 | Mm.260643 | Ywhaz | 3.15 | 2,08537 |
| BG074954 | Mm.197280 | Nr2c2 | 3.15 | 2,04957 |
| BG066006 | Mm.25743 | Tmprss2 | 3.14 | 2,33231 |
| BG067543 | Mm.258286 | Sp100 | 3.13 | 2,01155 |
| BG067807 | Mm.29373 | Mmp23 | 3.12 | 2,67757 |
| BG070560 | Mm.220901 | Lpgat1 | 3.12 | 2,48754 |
| BG075474 | Mm.148155 | Mod1 | 3.12 | 2,69955 |
| BG076253 | Mm.29823 | Mgst3 | 3.11 | 2,48683 |
| BG068718 | Mm.1921 | Fryl | 3.10 | 2,08242 |
| BG071707 | Mm.259949 | Slc6a8 | 3.10 | 2,74026 |
| BG074067 | Mm.28083 | Cd164 | 3.08 | 2,05864 |
| BG071644 | Mm.3786 | Slc34a2 | 3.07 | 2,23328 |
| BG071673 | Mm.26888 | Camk1d | 3.05 | 2,10487 |
| BG074507 | Mm.4394 | Kit | 3.03 | 2,03778 |
| BG067642 | Mm.24584 | Tmem49 | 2.99 | 2,28150 |
| BG064110 | Mm.27764 | Rnf128 | 2.99 | 2,10969 |
| BG074800 | Mm.173718 | Gtl6 | 2.99 | 2,00323 |
| BG070225 | Mm.196382 | Rsnl2 | 2.99 | 2,86828 |
| BG073604 | Mm.14796 | Mgst1 | 2.96 | 2,15358 |
| BG075145 | Mm.183034 | Stk38 | 2.95 | 2,00919 |
| BG071101 | Mm.27832 | Ralb | 2.91 | 2,05288 |
| BG068674 | Mm.26378 | Tesc | 2.91 | 3,22152 |
| BG075016 | Mm.30713 | Socs6 | 2.90 | 2,25740 |
| BG071728 | Mm.12915 | Nit1 | 2.89 | 2,57440 |
| BG075920 | Mm.126525 | Dagk | 2.88 | 2,02924 |
| BG067012 | Mm.1639 | Mcl1 | 2.87 | 2,32342 |
| BG072263 | Mm.1639 | Mcl1 | 2.86 | 2,32033 |
| BG073809 | Mm.2608 | Bgn | 2.85 | 2,33563 |
| BG069187 | Mm.6958 | Capn2 | 2.84 | 2,14908 |
| BG074814 | Mm.370 | C1qa | 2.83 | 2,98528 |
| BG074366 | Mm.243085 | Etv6 | 2.81 | 2,32457 |
| BG071506 | Mm.27917 | Tanc1 | 2.79 | 2,03085 |
| BG072227 | Mm.21119 | Litaf | 2.78 | 2,16970 |
| BG071169 | Mm.439733 | Camk2b | 2.72 | 2,45339 |
| BG075625 | Mm.22179 | Ergic2 | 2.71 | 2,00587 |
| BG075073 | Mm.142729 | Tmsb4x | 2.68 | 2,00811 |
| BG064913 | Mm.154286 | Ralgps2 | 2.67 | 2,17799 |
| BG071456 | Mm.57225 | Gpx2 | 2.65 | 3,19882 |
| BG067321 | Mm.28262 | Rgs2 | 2.64 | 2,25183 |
| BG074621 | Mm.239470 | Abca3 | 2.60 | 2,11436 |
| BG071381 | Mm.135621 | Ncald | 2.59 | 2,13839 |
| BG070686 | Mm.3863 | Por | 2.59 | 2,14565 |
| BG073108 | Mm.22506 | G7e | 2.57 | 6,43094 |
| BG070117 | Mm.43957 | Bxdc1 | 2.55 | 2,93736 |
| BG076077 | Mm.41078 | Wdr72 | 2.53 | 2,58964 |
| BG069051 | Mm.28034 | Plcl2 | 2.50 | 2,01685 |
| BG069421 | Mm.29133 | Bub1b | 2.49 | 2,67433 |
| BG067192 | Mm.30217 | Cib1 | 2.49 | 2,07899 |
| BG074809 | Mm.28099 | Soat1 | 2.43 | 2,11402 |
| BG071465 | Mm.259998 | Igf1 | 2.43 | 3,44328 |
| BG075595 | Mm.38387 | Qk | 2.43 | 2,01164 |
| BG076042 | Mm.182855 | Cbara1 | 2.43 | 2,70131 |
| BG070255 | Mm.5034 | Pde7a | 2.42 | 2,04881 |
| BG063978 | Mm.18626 | Capg | 2.41 | 2,02424 |
| BG076032 | Mm.29254 | Igfbp3 | 2.40 | 2,02024 |
| BG069253 | Mm.140761 | Dnajc5 | 2.36 | 2,09158 |
| BG067845 | Mm.206775 | Oas1c | 2.34 | 2,00554 |
| BG064710 | Mm.22478 | Smarcf1 | 2.31 | 2,49634 |
| BG064661 | Mm.29586 | Basp1 | 2.28 | 2,56368 |
| BG075814 | Mm.25227 | Klhl24 | 2.28 | 2,35980 |
| BG074327 | Mm.147387 | Col3a1 | 2.27 | 2,18133 |
| BG070107 | Mm.25880 | LOC218453 | 2.25 | 2,70188 |

# Significant downregulated genes

| Accesion Code | Unigene Number | Gene Name | Score(d) | Fold Change |
| --- | --- | --- | --- | --- |
| BG076333 | Mm.443 | Mthfd2 | -6.66 | 0,20999 |
| BG072212 | Mm.17655 | Hs3st3b1 | -6.59 | 0,13937 |
| BG064480 | Mm.192991 | Mt1 | -6.31 | 0,19619 |
| BG072125 | Mm.173903 | Sntb1 | -6.01 | 0,22808 |
| BG071962 | Mm.29622 | Ccdc16 | -5.48 | 0,18301 |
| BG064958 | Mm.227925 | Csrp2bp | -5.08 | 0,41595 |
| BG067541 | Mm.116862 | Mcoln2 | -5.03 | 0,17331 |
| BG076017 | Mm.2942 | Asns | -4.98 | 0,27563 |
| BG072041 | Mm.78861 | Nolc1 | -4.85 | 0,27603 |
| BG074372 | Mm.17403 | GlyRS | -4.53 | 0,35065 |
| BG065320 | Mm.196135 | Gemin6 | -4.43 | 0,36424 |
| BG067706 | Mm.200423 | Gpt2 | -4.41 | 0,28800 |
| BG067809 | Mm.27307 | Gng3 | -4.33 | 0,38194 |
| BG076068 | Mm.4952 | Irs1 | -4.16 | 0,35708 |
| BG066832 | Mm.172850 | Abcc1 | -4.06 | 0,43383 |
| BG070042 | Mm.104920 | Sdh1 | -4.02 | 0,35005 |
| BG071862 | Mm.56915 | Csf3r | -4.02 | 0,41670 |
| BG069760 | Mm.206417 | Cbs | -4.01 | 0,33962 |
| BG074462 | Mm.646 | Tpm2 | -4.00 | 0,33178 |
| BG071147 | Mm.200423 | Gpt2 | -4.00 | 0,29977 |
| BG073164 | Mm.258010 | Pgm2l1 | -3.98 | 0,26260 |
| BG072435 | Mm.32041 | Pgm2l1 | -3.96 | 0,28198 |
| BG063880 | Mm.255729 | Slc30a2 | -3.96 | 0,20991 |
| BG064735 | Mm.6587 | Prdx5 | -3.94 | 0,41365 |
| BG069752 | Mm.206417 | Cbs | -3.94 | 0,29896 |
| BG066347 | Mm.172736 | Wnk2 | -3.88 | 0,48940 |
| BG074541 | Mm.24276 | C1r | -3.82 | 0,21732 |
| BG065084 | Mm.4876 | Rcn | -3.80 | 0,31595 |
| BG071189 | Mm.29902 | Psat-pending | -3.79 | 0,45440 |
| BG063304 | Mm.30250 | Aldh7a1 | -3.76 | 0,48219 |
| BG064323 | Mm.7819 | Pld1 | -3.74 | 0,42185 |
| BG074268 | Mm.2011 | Gstm1 | -3.73 | 0,39590 |
| BG069852 | Mm.221029 | Fastkd1 | -3.67 | 0,46734 |
| BG069444 | Mm.24128 | C1s | -3.66 | 0,31526 |
| BG074546 | Mm.182726 | Dock9 | -3.61 | 0,38908 |
| BG075740 | Mm.39038 | Itpk1 | -3.55 | 0,37387 |
| BG067514 | Mm.39038 | Itpk1 | -3.52 | 0,43114 |
| BG070290 | Mm.154307 | Ift20 | -3.52 | 0,45908 |
| BG074397 | Mm.2011 | Gstm1 | -3.48 | 0,41030 |
| BG063736 | Mm.9001 | Nlk | -3.42 | 0,45846 |
| BG065196 | Mm.4419 | Rpl5 | -3.39 | 0,49496 |
| BG065154 | Mm.18737 | Kntc1 | -3.37 | 0,47502 |
| BG067972 | Mm.25530 | Plekha4 | -3.36 | 0,49303 |
| BG066852 | Mm.153315 | Atf6 | -3.34 | 0,42313 |
| BG073197 | Mm.27944 | Acsl3 | -3.33 | 0,38014 |
| BG069966 | Mm.203125 | Pan3 | -3.32 | 0,32431 |
| BG073280 | Mm.33650 | Pycr1 | -3.31 | 0,47403 |
| BG066941 | Mm.10 | Srm | -3.24 | 0,38187 |
| BG063119 | Mm.16972 | Chd7 | -3.16 | 0,40761 |
| BG070044 | Mm.27680 | Mylk | -3.14 | 0,34409 |
| BG065409 | Mm.155620 | Pla2g6 | -3.12 | 0,44252 |
| BG071956 | Mm.18652 | Sms | -3.12 | 0,46322 |
| BG072053 | Mm.180553 | Nr2e1 | -3.04 | 0,38362 |
| BG067357 | Mm.4356 | Cnn1 | -2.88 | 0,42385 |
| BG069668 | Mm.104920 | Sdh1 | -2.87 | 0,43166 |
| BG075348 | Mm.24210 | Bcat2 | -2.80 | 0,37178 |
| BG075019 | Mm.143603 | Sh3gl2 | -2.75 | 0,49452 |
| BG063438 | Mm.28665 | Edem2 | -2.71 | 0,45748 |
| BG065337 | Mm.34388 | Nudt16l1 | -2.70 | 0,49022 |
| BG074523 | Mm.25848 | Bckdha | -2.65 | 0,46688 |
| BG071284 | Mm.23352 | Suox | -2.65 | 0,45828 |
| BG069993 | Mm.200423 | Gpt2 | -2.64 | 0,45515 |
| BG063884 | Mm.196080 | Wdr42a | -2.60 | 0,47434 |
| BG066444 | Mm.38154 | Bcas3 | -2.59 | 0,46205 |
| BG072362 | Mm.42255 | Atp2a2 | -2.58 | 0,42757 |
| BG066796 | Mm.16898 | Phgdh | -2.57 | 0,44819 |
| BG072224 | Mm.138100 | Tssc8 | -2.53 | 0,43942 |
