# Supplementary material for: Accumulating Progenitor Cells in the Luminal Epithelial Cell Layer Are Candidate Tumor Initiating Cells in a Pten Knockout Mouse Prostate Cancer Model
Source: PLoS One. 2009 May 22;4(5):e5662. doi: 10.1371/journal.pone.0005662 (PMC2680948; doi:10.1371/journal.pone.0005662)
Supplement: Table S3 — Primer sequences of genes analyzed by QPCR. (0.03 MB DOC) [file pone.0005662.s008.doc]

**Table S3. Primer sequences of genes analyzed by QPCR.**

| **Gene name** | **Forward primer** | **Reverse primer** |
| --- | --- | --- |
| Hprt | 5’-TCCCTGGTTAAGCAGTACAG-3’ | 5’-TTCCAGTTTCACTAATGACAC-3’ |
| Nkx.3.1 | 5’-ACTGAACCCGAGTCTGATGC-3’ | 5’-CTTGGGTTTCGGTGAGTTTG-3’ |
| Probasin | 5’-ACAACTGTCCAAGCAAGATC-3’ | 5’-TGATGTTTCAGGTTCCAGGA-3’ |
| CK8 | 5’-CTCCGGCAGATCCATGAAG-3’ | 5’-GGTACATGGTTTCAGCCTC-3’ |
| CK19 | 5’-GGGCCTTGAGATTGAGCTGC-3’ | 5’-GGTTCTGGCGCTCTATGTCG-3’ |
| CK5 | 5’-CAGGACATGGCCAGGCTG-3’ | 5’-CAACTCCTTCCCCACTCAGC-3’ |
| p63 | 5’-CCCACAGACTGCAGCATTG-3’ | 5’-GAGATGAGGAGGTGAGGAGAAG-3’ |
| Expi | 5’-GGAGATGGATCGTGCTCTGG-3’ | 5’-GGCTAGCCATCAGTCCTGC-3’ |
| Wfdc2 | 5’-TGTGACCAGGGAAGGCTTAGG-3’ | 5’-CTCCAGATGCACAGTCCGGC-3’ |
| Tacstd2 | 5’-GACCTCTTCCTTCTCTCTCACC-3’ | 5’-CAAGTCCCTGGGAAACAAGTG-3’ |
| Clu | 5’-GTGAAGCTGTTTGACTCTGACC-3’ | 5’-GATTCCCTCCCAGACACTCC-3’ |
| Ppp1r1b | 5-CTGAGGACCAAGTGGAAGGC-3’ | 5’-CAGGGTACAAAGGAGGGTGG-3’ |
| Sca-1 | 5’-GTCCCATTTGAGACTTCTTGCC-3’ | 5’-AGGAGGGCAGATGGGTAAGC-3’ |
| Hs3st31b | 5’-CCAGTCCCATCTCCAGCTTC-3’ | 5’-GGCATCAAGTCTCGGTACCAG-3’ |
| Ccdc16 | 5’-TGCTCTGTAGTTTACTGTACTCC-3’ | 5’-GCAACTTACCTATCTTCCTGCC-3’ |
| Mcoln2 | 5’-GGAGAGCGGAAGCAAAGATGG-3’ | 5’-ACCTCTGCATAAAGGGATCTGG-3’ |
| C1r | 5’-TGGAAGAATGAAGAGGAAGGAG-3’ | 5’-CGTGGGTAGTGGTGAAGGC-3’ |
| Mt1 | 5’-CTCCAGCTTCACCAGATCTCG-3’ | 5’-CCTTTGCAGACACAGCCC-3’ |
