# Supplementary material for: Accumulating Progenitor Cells in the Luminal Epithelial Cell Layer Are Candidate Tumor Initiating Cells in a Pten Knockout Mouse Prostate Cancer Model
Source: PLoS One. 2009 May 22;4(5):e5662. doi: 10.1371/journal.pone.0005662 (PMC2680948; doi:10.1371/journal.pone.0005662)
Supplement: Table S4 — Information of antibodies used for immunohistochemistry and immunofluorescence (0.03 MB DOC) [file pone.0005662.s009.doc]

**Table S4. Information of antibodies used for immunohistochemistry and immunofluorescence**

| Antibody | **Company** | **Product number** | **Method** | **Dilution** |
| --- | --- | --- | --- | --- |
| **CK8** | Covance, Berkeley, CA | MMM-162P | IHC | 1:1000 |
|  |  |  | IF | 1:1000 |
| **CK19** | Abcam, Cambridge, UK | ab15463 | IHC | 1:200 |
| **Nkx3.1** | -- | -- | IHC | 1:1000 |
| **p63** | DAKO | M7247 | IHC | 1:100 |
|  |  |  | IF | 1:50 |
| **Tacstd2** | R&D systems, Minneapolis, MN | AF1122 | IHC | 1:100 |
|  |  |  | IF | 1:100 |
| Clu | Santa Cruz, CA | SC-6420 | IHC | 1:800 |
|  |  |  | IF | 1:400 |
| Ppp1r1b | Cell Signaling Technology, Beverly, MA | 2302 | IHC | 1:100 |
| **Sca-1** | R&D systems, Minneapolis, MN | AF1226 | IHC | 1:100 |
| **pAkt** | Cell Signaling Technology, Beverly, MA | 4058 | IHC | 1:100 |
|  |  |  | IF | 1:50 |
| **Goat Anti-Mouse-biotin** | DAKO | E0433 | IHC | 1:400 |
| **Swine Anti-Rabbit-biotin** | DAKO | E0431 | IHC | 1:400 |
| Rabbit Anti-Goat-biotin | Santa Cruz, CA | SC-2774 | IHC | 1:400 |
|  |  |  | IF | 1:400 |
| Streptavidin-TRITC | Invitrogen, Carlsbad, CA | 43-4314 | IF | 1:50 |
| **Rabbit Anti-Mouse-FITC** | DAKO | F0261 | IF | 1:40 |
| **Rabbit Anti-Mouse-TRITC** | DAKO | R0270 | IF | 1:100 |
| **Swine Anti-Rabbit-FITC** | DAKO | F0205 | IF | 1:100 |

IHC: Immunohistochemistry

IF: immunofluorescence
